# Interspecific introgression mediates adaptation to whole genome duplication

**DOI:** 10.1038/s41467-019-13159-5

**Published:** 2019-11-18

**Authors:** Sarah Marburger, Patrick Monnahan, Paul J. Seear, Simon H. Martin, Jordan Koch, Pirita Paajanen, Magdalena Bohutínská, James D. Higgins, Roswitha Schmickl, Levi Yant

**Affiliations:** 1 Department of Cell and Developmental Biology, John Innes Centre, Norwich, NR4 7UH UK; 2 Department of Genetics and Genome Biology, University of Leicester, Adrian Building, University Road, Leicester, LE1 7RH UK; 3 Institute of Evolutionary Biology, University of Edinburgh, Edinburgh, EH9 3FL UK; 4 Department of Botany, Faculty of Science, Charles University, Benátská 2, 128 01 Prague, Czech Republic; 5 The Czech Academy of Sciences, Zámek 1, 252 43 Průhonice, Czech Republic; 6 Future Food Beacon of Excellence and the School of Life Sciences, University of Nottingham, Nottingham, UK

**Keywords:** Evolutionary genetics, Polyploidy in plants, Plant evolution, Genome duplication

## Abstract

Adaptive gene flow is a consequential phenomenon across all kingdoms. Although recognition is increasing, there is no study showing that bidirectional gene flow mediates adaptation at loci that manage core processes. We previously discovered concerted molecular changes among interacting members of the meiotic machinery controlling crossover number upon adaptation to whole-genome duplication (WGD) in *Arabidopsis arenosa*. Here we conduct a population genomic study to test the hypothesis that adaptation to WGD has been mediated by adaptive gene flow between *A. arenosa* and *A. lyrata*. We find that *A. lyrata* underwent WGD more recently than *A. arenosa*, suggesting that pre-adapted alleles have rescued nascent *A. lyrata*, but we also detect gene flow in the opposite direction at functionally interacting loci under the most extreme levels of selection. These data indicate that bidirectional gene flow allowed for survival after WGD, and that the merger of these species is greater than the sum of their parts.

## Abstract (web summary)

Whole genome duplication (WGD) presents new challenges to the establishment of optimal allelic combinations and to the meiotic machinery. Here, the authors show that adaptive gene flow from *Arabidopsis arenosa* could rescue the nascent *A. lyrata* from extinction following WGD.

## Introduction

Whole-genome duplication (WGD) and hybridisation are key drivers of genomic novelty, promoting diversification in all kingdoms of life^1–3^. Recent progress in evolutionary genomics underscores the high frequency of WGD at both ancient and recent time scales^4^, and population genomic approaches reveal widespread evidence of gene flow between the most diverse species^5,6^. Both processes have therefore been associated with adaptive benefits. However, WGD and hybridisation are large-effect mutations, often leading to a host of genomic instabilities, including epigenetic shock, perturbed gene expression patterns and meiotic instability, with direct negative impacts on fertility. Perhaps the most challenging issue is the most immediate: that of stable meiotic chromosome segregation following WGD. How nascent polyploids establish meiotic stabilisation remains an unresolved question.

The wild outcrossing members of the *Arabidopsis* genus have recently emerged as fruitful models for the study of genome stabilisation following WGD^7^. *Arabidopsis arenosa* is a largely biennial outcrossing relative of the model *Arabidopsis thaliana*, which forms distinct lineages of diploids and autotetraploids throughout Central Europe^8–11^. Initial resequencing of a handful of autotetraploid *A. arenosa* individuals suggested selective sweep signatures at genes involved in genome maintenance, including DNA repair, recombination and meiosis^12^. Later, a targeted resequencing effort focused on patterns of differentiation between diploid and autotetraploid *A. arenosa*, revealing evidence of highly localised selective sweeps directly overlapping eight loci whose gene products interact during prophase I of meiosis^13^. These eight loci physically and functionally interact to control crossover designation and interference, strongly implying that a modulation of crossover distribution was essential for polyploid establishment in *A. arenosa*^14,15^. Cytological evidence of a reduction in crossover numbers in the autotetraploids indicated that the selected alleles had an effect^13^. Similar to its sister species *A. arenosa* (*arenosa* hereafter), *Arabidopsis lyrata* (*lyrata* hereafter) also naturally occurs as diploids and tetraploids across its distribution range^8,16–18^. Although there is little evidence for gene flow among diploids of each species, there have been reports of gene flow between tetraploid *arenosa* and *lyrata* and, less pronounced, gene flow between diploids and tetraploids^8,19,20^.

Here we investigate the molecular basis of parallel adaptation to WGD in *lyrata* compared with *arenosa* and the possibility of adaptive gene flow between the two species. Specifically, we ask (1) whether the same or different loci may be involved in adaptation to WGD in *lyrata* as we observed in *arenosa*; and (2) whether these adaptations arose independently or via introgression from one species into the other. Using whole-genome sequence data from 92 individuals of *lyrata*, *arenosa* and outgroup species *Arabidopsis croatica* and *Arabidopsis halleri*, we first analyse population structure and demography, concentrating on assessing admixture and the degree and timing of population divergences. Then, to estimate the relative degree of adaptation to WGD across the ranges of *lyrata* and *arenosa*, we cytologically assess meiotic stability in key populations. Finally, after scanning the *lyrata* genomes for signatures of selective sweeps, we compare the most differentiated regions with those we previously found in *arenosa*^12,13^ and test whether these selective sweep signatures overlap with fine-scale conspicuous introgression signals. Overall, our results reveal the molecular basis by which WGD has been stabilised in both species and indicate that WGD-facilitated hybridisation allowed for stabilisation of meiosis in nascent autotetraploids by highly specific, bidirectional adaptive gene flow.

## Results

### Population structure and broad-scale admixture

**Fig. 1:**
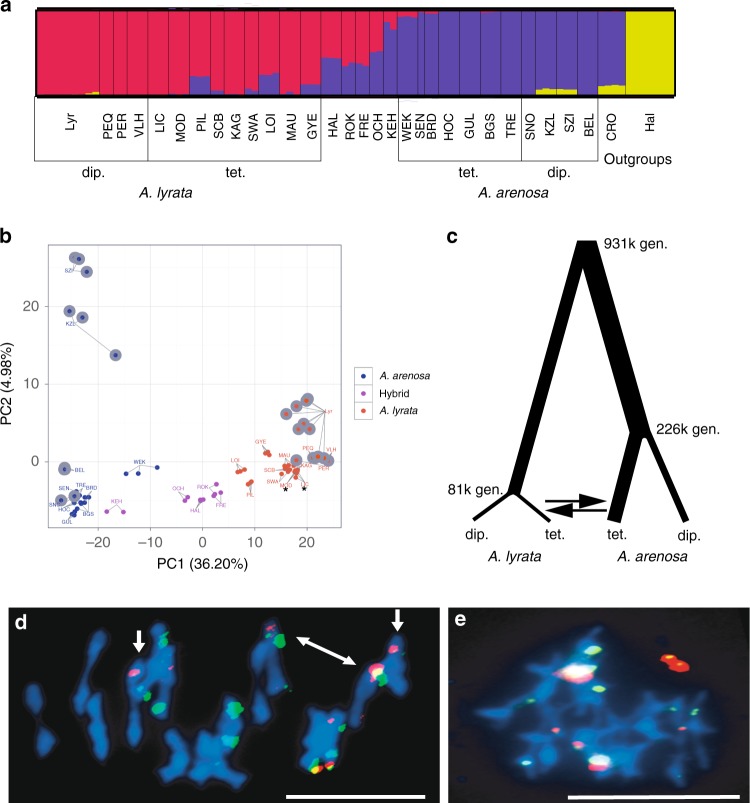
Ploidy-specific admixture and stable autotetraploid meiosis in *A. lyrata*. **a** A continuous range of admixture specifically in tetraploid populations demonstrated with STRUCTURE analysis of nuclear SNP data (32,256 LD-pruned, 4-fold degenerate SNPs). Populations (in three-letter code) and population groupings (ploidy, species) are displayed. Populations are described in (Supplementary Table 1). **b** PCA shows individuals group on the main (PC1) axis by species and not by ploidy, with hybrid individuals located between *A. lyrata* and *A. arenosa* samples. We refer to all non-pure populations from the hybrid zone in the eastern Austrian Forealps as hybrids (see Supplementary Fig. 1). Diploids are indicated by grey outline. Asterisks (*) are placed under the *Let* tetraploid grouping; all other *A. lyrata* tetraploids (except the geographically divergent Pannonian GYE) are in the *Lwt* group. **c** Demographic parameter estimates for *A. lyrata* and *A. arenosa* populations. Line widths are proportional to estimates given in Supplementary Fig. 2. **d**, **e** Metaphase I chromosome spreads of nuclei from two ROK plants hybridised with 5S rDNA (red) and 45S rDNA (green). **d** MI scored as stable as 16 individual bivalents are observed, even though there are bivalents with unequal probes (white arrows), suggesting non-homologous rearrangements. **e** MI scored as unstable as the majority of chromosomes are connected to each other. Chromosomes are stained with DAPI; bar = 10 µm. The source data underlying Fig. 1d, e are provided as a Source Data file

To understand population and species relationships, we analysed the genomes of 92 individuals from ~30 populations of *lyrata* and *arenosa* throughout Central Europe along with outgroups, sequenced at a depth of ~15× per individual (Supplementary Table 1 and Supplementary Fig. 1). STRUCTURE and principal component analysis (PCA) showed a clear species-specific clustering for diploids, whereas tetraploids exhibited a gradient of relatedness between species (Fig. 1a, b). Admixture was markedly lower in *arenosa* populations than in *lyrata*: consistently, all diploids tested (SNO, KZL, SZI, BEL) and tetraploids from the Western Carpathians (TRE), and most of the Alpine tetraploids (HOC, GUL, BGS) harboured essentially pure *arenosa* genomes (Fig. 1a). Minimal admixture signal (<1%) with *arenosa* was detected in a few *lyrata* genomes, in particular the Austrian diploid (PEQ, PER, VLH), as well as the *lyrata* eastern tetraploid (*Let* hereafter) populations (LIC, MOD) and the tetraploid KAG population (Fig. 1a).

In contrast, many other *lyrata* populations exhibited substantial admixture signals with *arenosa*, varying drastically in degree (Fig. 1a). Several tetraploid *lyrata* populations from the Wachau (SCB, SWA, MAU) displayed only slight admixture with *arenosa* and populations at the Wachau margin (PIL, LOI) showed stronger admixture, probably due to the increased proximity to the Hercynian and Alpine *arenosa* lineages^21^. Compared with the Wachau, where *lyrata* occurs on the slopes and hilltops along the Danube river surrounded by *arenosa* populations outside of the valley, there is a classical hybrid zone in the eastern Austrian Forealps: the parental species are found at the two poles of the zone (diploid and tetraploid *lyrata* in the Wienerwald, tetraploid *arenosa* at higher altitudes to the west and the hybrids between; Supplementary Fig. 1). Populations HAL, ROK, FRE, OCH and KEH are heavily admixed, with KEH appearing more *arenosa*-like, and ROK and FRE being slightly more *lyrata*-like compared with the others (Fig. 1a). Again, proximity of the hybrids to the donor species corresponded with increased admixture. The Hungarian tetraploid *lyrata* population GYE also exhibited admixture signal, suggesting that gene flow between *lyrata* and *arenosa* is not restricted to the Austrian Forealps. PCA was consistent with STRUCTURE findings, with PC1 dividing samples by species (explaining >36% of the variance; Fig. 1b). The second axis (<5% of the variance) separated KZL and SZI from the other diploid *arenosa* populations. These are representatives of the Pannonian lineage, which is the oldest and most distinct diploid *arenosa* lineage^21^. Overall, our results are consistent with previous descriptions of introgression between *lyrata* and *arenosa* in Austria that were based on smaller marker sets and different sampling schemes^8,22^.

We estimated the population split time without migration between *lyrata* and *arenosa* at 931,000 (931k) generations ago using *fastsimcoal2*^23^ (Fig. 1c, Supplementary Figs. 2 and 3, and Supplementary Table 2). This translates to ~2 million years ago (mya), given an average generation time of 2 years, which would coincide with the onset of Pleistocene climate oscillations. This estimate lies within the range of age estimates for this split from ref. ^24^ with 1.3 mya and from ref. ^25^ with 8.2 mya, and ref. ^22^. We estimated the age of WGDs at 81k generations ago for *lyrata* (~160,000 years ago) and 226k generations ago for *arenosa* (~450,000 years ago), which approximately mark periods of glacial maxima^26^. Noting this, we next asked if either species experienced substantial historical bottlenecks. Using pairwise sequentially Markovian coalescent (PSMC) model^27^ we could not reach ages as ancient as 130–300 kya (Supplementary Fig. 4), because the recombinant blocks that PSMC measures are too short in these diverse outcrossing species to estimate ancient population histories. Our analysis indicated that diploid *lyrata* had a peak effective population size (*Ne*) ~25 kya (PER, VHL) and ~20 kya (PEQ), whereas diploid Dinaric *arenosa* (BEL) peaked earlier, at ~30 kya. Interestingly, diploid Western Carpathian *arenosa* SNO, the population that founded several widespread autotetraploid lineages^9^, gave a strong signal of continuous expansion. These results suggest that diploid *lyrata* and partly *arenosa* underwent a bottleneck after the last glacial maximum 30–19 kya. PSMC does not accommodate autotetraploid data, but using *fastsimcoal2* we detected a strong bottleneck at WGD for *lyrata* and none for *arenosa* (Supplementary Fig. 2).

We next assayed for patterns of gene flow using coalescent modelling with *fastsimcoal2*. Due to model overfitting when using more than two migration edges, we chose the model retaining only two migration edges with the highest support: interspecific gene flow from tetraploid *arenosa* to tetraploid *lyrata* (0.1 alleles/generation) and *lyrata* to *arenosa* gene flow at the same level (0.1 alleles/generation) (Fig. 1c, Supplementary Figs. 2 and 3, and Supplementary Table 2). These results indicate equal amounts of bidirectional gene flow specifically among the tetraploids of both species.

### Stabilisation of *lyrata* meiosis following WGD

Given the very low abundance of *lyrata* tetraploids compared with tetraploid *arenosa* in nature, we assayed whether these tetraploids were indeed meiotically stable. Cytological analysis indicated that in fact *lyrata* tetraploids exhibit similar levels of meiotic stability as *arenosa* tetraploids, as evidenced by relative percentages of stable rod and ring bivalents (Fig. 1d and Supplementary Table 3) vs. less stable multivalents (Fig. 1e and Supplementary Table 3). We were surprised to observe among both species that meiotic stability segregates within populations, typically ranging from <20 to >60% stable metaphase I cells per plant with extremes observed in KAG (0–98%) and consistently higher levels (>80%) in the *arenosa* populations. Meiotic stability was also variable within the tetraploid *arenosa* population TBG, which was the population used by Yant et al.^13^ to cytologically assess meiotic stability. A much higher number of chromosome spreads on more individuals and populations in the present study indicates that meiosis is not universally stable among autotetraploids across these populations. Overall, these results indicate that meiotic stability is broadly segregating within tetraploid populations of both species.

### Selective sweep signatures in *lyrata*

To gain insight into the processes underlying adaptation to WGD in *lyrata* tetraploids, we performed a population-based genome scan for selection. We quantified differentiation between *lyrata* ploidies by calculating *d*_*XY*_^28^, Fst^29^ and Rho^30^ in adjacent windows along the genome between diploids and tetraploids. Fst is influenced by within-population diversity and lacks sensitivity in cases of low differentiation. Therefore, we used additional differentiation metrics. *d*_*XY*_ does not take within-population diversity into account, whereas Rho is a divergence measure that is independent of ploidy level and double reduction in autopolyploids. We focused on the non-admixed *lyrata* tetraploid populations LIC and MOD (*lyrata* eastern tetraploids; *Let*), which by STRUCTURE and PCA analyses exhibited the lowest levels of admixture (Fig. 1a) and clustered with *lyrata* diploids, distant from the *arenosa* tetraploids or the broadly admixed *lyrata* tetraploids (Fig. 1b). Overall, genome-wide differentiation levels between *lyrata* diploids and the tetraploids indicate shallow divergence between all groups (with mean Rho in the most differentiated contrast between ploidies = 0.19; Table 1 and Supplementary Table 4 for additional population contrasts), consistent with our previous studies in *arenosa*^12,13,21,31^.

**Table 1:** Genome-wide differentiation between *A. lyrata* diploids and tetraploids, and between tetraploid lineages grouped by biogeography

Contrast	No. of SNPs	AFD	*d*_*XY*_	Fst	Rho	Fixed Diff
Diploid *lyrata* vs. *lyrata* eastern tetraploids (*Let*)	2,904,110	0.14	0.22	0.09	0.19	270
Diploid *lyrata* vs. *lyrata* Wachau tetraploids (*Lwt*)	3,794,257	0.11	0.16	0.07	0.17	64
*Lyrata Let* tetraploids vs. *Lwt* tetraploids	4,795,381	0.09	0.16	0.06	0.13	24
*Arenosa* Hercynian tetraploids vs. *arenosa* Alpine tetraploids	1,812,223	0.10	0.16	0.03	0.07	0

Differentiation metrics shown are allele frequency difference (AFD), *d*_*XY*_, Fst, Rho and the number of fixed differences (Fixed Diff). Multiple differentiation metrics were used, as the metrics exhibit different sensitivities to diversity and differentiation. Values of all metrics were averaged over pairwise comparisons of populations belonging to that group

To identify the most robust signals of selection in the tetraploid *lyrata* populations, we performed genome scans on two different *lyrata* tetraploid population groups and then focussed on the genes that were repeatedly in the extreme 1% outlier windows in both contrasts. This identified 196 genes (0.6% of gene-coding loci in the genome; Supplementary Dataset 1). First, contrasting the *lyrata* diploids and the *Let* tetraploids, we partitioned the genome into gene-sized windows and identified outliers for allele frequency differences (AFDs), *d*_*XY*_, Fst, Rho and the number of fixed differences. Although the comparison of the most pure *lyrata* tetraploid populations, represented by the *Let* group, to *lyrata* diploids is the most stringent test of which loci are under selection in a purely *lyrata* genomic context, we extended our tetraploid *lyrata* sampling to populations from the Wachau, which frequently showed admixture with *arenosa* (*lyrata* Wachau tetraploids, *Lwt* hereafter: PIL, SCB, KAG, SWA, LOI and MAU; GYE was excluded due to distant geographic grouping in Pannonia). As the *Let* and part of the *Lwt* populations grow in contrasting edaphic conditions (*Let* on limestone, *Lwt* on siliceous bedrock), we used this approach to maximise our chances of capturing differentiation specifically related to ploidy and not local adaptation. In addition, we observed that differentiation between these two tetraploid *lyrata* groups is stronger than differentiation between the tetraploid *arenosa* lineages studied here (Table 1), suggesting that there is stronger genetic structure within *lyrata* than *arenosa*, as was observed by ref. ^32^, and supporting a degree of independence between the *Let* and *Lwt* divergence scans.

Gene Ontology (GO) enrichment analysis of these 196 genes identified significant overrepresentations in categories related to meiotic and homologous chromosome segregation, but also diverse processes including epidermal cell differentiation, trichoblast maturation, root hair cell and epidermal differentiation, root hair cell development and elongation, and others such as indole-containing compound metabolic process and mRNA catabolic process (Supplementary Fig. 5 and Supplementary Dataset 2). These results indicate that evolutionary change may occur throughout a broad array of processes during adaptation to WGD, beyond meiotic chromosome segregation.

**Fig. 2:**
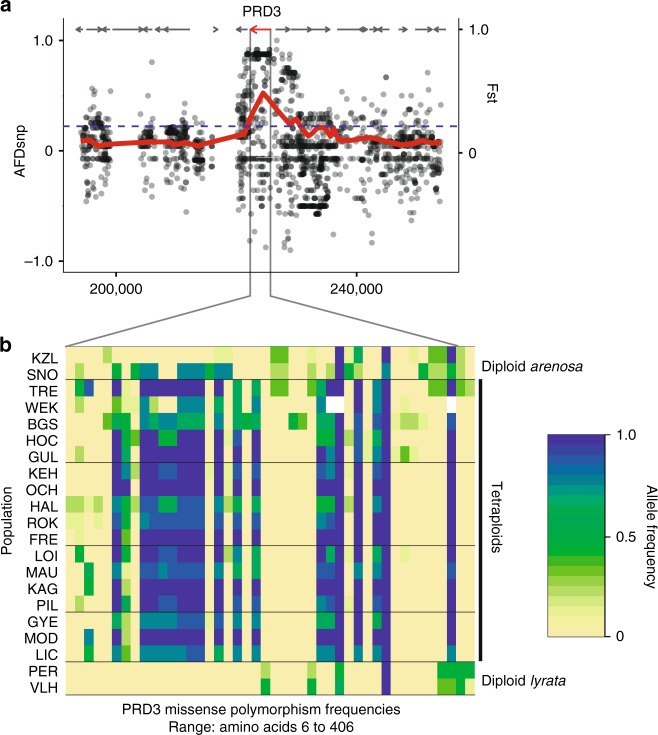
Selective sweeps and missense polymorphism frequencies by population. **a** Selective sweep example in *PRD3*, a gene involved in meiotic double strand break formation. *X*-axis gives chromosome 1 position in base pairs. Left *Y*-axis gives allele frequency differences between diploid and tetraploid *A. lyrata* and at single-nucleotide polymorphisms (dots). Right *Y*-axis (and red line) gives local Fst. Arrows indicate gene models. Red arrow indicates selective sweep candidate with localised differentiation. The dotted line gives the 99th percentile of genome-wide Fst values. **b** Zoom-in on *PRD3* coding changes. Heatmap represents allele frequencies of missense polymorphisms. Frequencies 0–100% follow yellow to green, to blue. Derived diploid *A. arenosa*-specific missense polymorphisms are driven to high frequency in the tetraploids, whereas diploid *A. lyrata* alleles are absent, implicating diploid *A. arenosa* origin to this selected allele in the tetraploids

Comparing this set of outliers to those found under selection upon WGD in *arenosa*^13^, 20 gene-coding loci exhibited the highest levels of differentiation in both studies (Table 2). These included those meiosis-related loci reported above (*PRD3*, *ASY1*, *ASY3* and *SYN1*), as well as the endopolyploidy genes *CYCA2;3* and *MEE22*, and the global transcriptional regulator *TFIIF*, among others. We observed selective sweep signatures at the majority (6/11) of coding loci of known function that were found as the very top outliers in *arenosa* (0.5% outliers for all three metrics used in that study) having primary functions of mediating meiosis, endopolyploidy and transcription. In particular, outlier loci participating in meiotic crossover formation, including *ASY1*, *ASY3*, *PDS5-like*, *PRD3*, and *SYN1* exhibited tight peaks of divergence directly over single gene-coding loci (an example is given in Fig. 2), a divergence signal we have broadly seen in this system^13,21,31^. In addition, the meiosis loci important for crossover formation reported by Yant et al.^13^ *ZYP1b* and *PDS5* were outliers in the *Lwt* contrast. The paralog *ZYP1b* was differentiated in the *Let* group also, but was not among the 1% top outliers; *PDS5* showed no differentiation between the *Let* and *lyrata* diploids. *SMC3*, a top outlier in *arenosa*, showed only moderate differentiation in the *Lwt* and no differentiation in the *Let* scan. Taking this most restrictive list representing the overlap of three genome scans, GO enrichment analysis identified significant overrepresentations only in categories related to meiotic chromosome segregation (Supplementary Dataset 3). These results further support the notion that these same loci were under the highest levels of selection following the more recent WGD event in *lyrata* as were under selection following the independent, earlier WGD (Fig. 1c) in *arenosa*.

**Table 2:** Overlap list of the top 1% outliers from the genome scans

*Lyrata* ID	Name	Description	*Let* scan Outlier	*Lwt* scan Outlier
AL1G10680	PRD3	Involved in meiotic double strand break formation	Yes	Yes
AL1G27690	CYCA2;3	Negatively regulates endocycles and acts as a key regulator of ploidy levels in endoreduplication	Yes	Yes
AL1G36300	PBP3	Putative poly(A) binding protein	Yes	Yes
AL2G25520	SWEETIE	Involved in trehalose metabolic process	Yes	Yes
AL2G25920	ASY1	ASYNAPTIC 1 mediates meiotic crossovers	Yes	Yes
AL2G37810	PDS5-like	ARM repeat superfamily protein	Yes	Yes
AL2G40680	CMT1	Chromomethylase 1 DNA methyltransferase	Yes	Yes
AL4G29630	NAB	Nucleic acid-binding, OB-fold-like protein	Yes	Yes
AL4G29650	Unknown		Yes	Yes
AL4G30770	MEE22	Involved in endoreduplication and cell fate	Yes	Yes
AL4G46460	ASY3	ASYNAPTIC 3 required for normal meiosis	Yes	Yes
AL5G13440	ASF	Asparagine synthase family protein	Yes	Yes
AL5G32850	PSF	Pseudouridine synthase family protein	Yes	Yes
AL5G32860	TFIIF	Functions in RNA polymerase II activity	Yes	Yes
AL5G32870	GTE6	Bromodomain containing nuclear-localised protein involved in leaf development	Yes	Yes
AL5G39280	NRPA1	Subunit of RNA polymerase I (aka Pol A)	Yes	Yes
AL6G15380	SYN1	A RAD21-like gene essential for meiosis	Yes	Yes
AL7G35790	unknown		Yes	Yes
AL8G25590	DYAD, SWI1	Involved in meiotic chromosome organisation	Yes	Yes
AL8G25600	TPR-like	Tetratricopeptide repeat (TPR) protein	Yes	Yes
AL1G35730	ZYP1a, b	Transverse filament of meiotic synaptonemal complex	No	Yes
AL4G20920	SMC3	Member of the meiotic cohesin complex	No	No
AL8G10260	PDS5	Member of the meiotic cohesin complex	No	Yes

Overlap list of the top 1% outliers from the genome scan of diploid *A. lyrata* vs. *Let* and diploid *A. lyrata* vs. *Lwt* overlapped with the outliers of the *A. arenosa* diploid–tetraploid scan of Yant et al.^13^. The overlap between the diploid/*Let* and diploid/*Lwt* contrasts yielded 196 genes, which is approximately a third of the genes identified in each scan. The overlap of those two scans with the *A. arenosa* scan gave 20 genes in common. Core meiosis genes found in Yant et al.^13^, which were found in only one or none of the two *lyrata* scans, are stated in the bottom part of this list

Apart from loci encoding meiosis-related genes, we detected extreme differentiation at loci belonging to other functional categories clearly related to the challenges attendant to WGD, including loci involved in endoreduplication and transcriptional regulation: *CYCA2;3*, *PAB3*, *NAB*, *TFIIF* and *GTE6*. WGD increases the ploidy of all cells, whereas endopolyploidy occurs in single cells during their differentiation, and this cell- and tissue-specific ploidy variation is important in plant development^33–35^. Thus, given the instantly doubled organism-wide nuclear content following WGD, we postulate that the degree of endopolyploidy would be modulated in response, with accumulating support for this notion^36–38^. Our findings bolster the idea that there may be a link between organism-wide polyploidization, and that of single cells within an organism. Research about the effect of WGD-induced dosage responses of the transcriptome is still in its infancy^3,39^ and emerging studies on allopolyploids support incomplete dosage compensation.

### Highly specific introgression at sweep genes

**Fig. 3:**
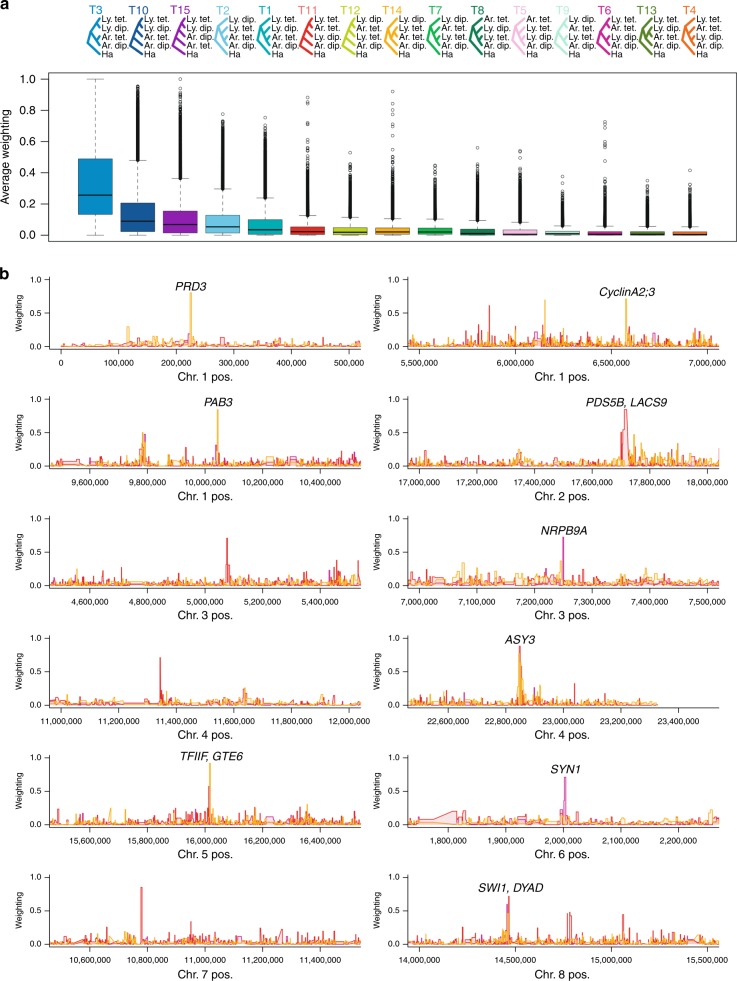
Highly specific introgression events across species boundaries. **a** Topologies from *Twisst* analysis of *Lwt*: Although topology 3 is the dominant species tree, topologies 11, 14 and 6 indicate localised gene flow between tetraploids. Box plots give relative weightings of all topologies across the genomes analysed. It is noteworthy that the extreme outliers concentrate specifically on the introgression-indicative topologies 11, 14 and 6. The bold line indicates the median. The box spans the first and third quartiles, and the whiskers extend to the most extreme point within 1.5 times the interquartile range from the box. Source data are provided as a Source Data file. **b** Introgression events revealed by *Twisst* analysis are highly localised at loci encoding genes controlling meiosis, endopolyploidy, and transcriptional control. All gene-coding loci under a given narrow peak are labelled; many of the indicated loci are divergence scan outliers in both the *Let* and *Lwt* divergence scans in addition to being Twisst outliers. The genome-wide dominant topology 3 weightings are omitted in **b** for clarity. The colours in **b** correspond to topologies 11, 14 and 6 in **a**. The weighting quantifies the extent to which each 50 SNP window tree matches a given topology, accounting for the fact that each taxon is represented my multiple individuals that each have 2 (for diploids) or 4 (for tetraploids) tips in the tree. A weighting of 1 indicates that all individuals cluster in the same way, such that all possible subtrees match the same topology. Weightings >0 but <1 indicate that different subtrees match different topologies. Source data are provided as a Source Data file

Finally, we sought to confirm whether the strong observed signals of selective sweep were the products of localised interspecific introgression. To confirm candidate introgressed regions at high resolution, we used *Twisst*^40^, performing two independent analyses, with either *Let* or *Lwt* representing tetraploid *lyrata*. The consensus species phylogeny, topology 3, represented the overwhelmingly dominant genome-wide topology (Fig. 3a). Topologies consistent with introgression (6, 11 and 14, which group tetraploids of the two species together) all had comparatively low values, but also showed multiple narrow peaks across the genome. Twelve peaks had weightings >0.7 and nine of these overlapped with our divergence scan outliers (Fig. 3b and Supplementary Dataset 4). Similarly, 61 had a weighting >0.5 and 21 (34%) of these overlapped with gene-coding loci that were positive in both the *Let* and *Lwt* divergence scans (Fig. 3b and Supplementary Dataset 4). This degree of overlap of the loci found under selection in our genome scans is dramatically greater than expected by chance (0.6%), which we confirmed by performing permutation tests (Supplementary Fig. 6). By contrast to the introgression-indicative topologies, those consistent with incomplete lineage sorting (ILS) alone (7, 8, 9, 12 and 13, which group diploid *arenosa* with tetraploid *lyrata* or vice versa) were low genome-wide with only two peaks reaching above 0.5 (Fig. 3a).

Similar to the divergence outlier windows, *Twisst*-positive windows were narrow, which might be an indication that genomic differentiation following divergence between *lyrata* and *arenosa* is advanced and introgression occurred fairly deep in the past, similar to the numerous narrow genomic regions of introgression in the case of gene flow between *Populus alba* and *Populus tremula*^41^. However, we have recently estimated linkage disequilibrium (LD) in this system^21^, finding a very rapid reduction specifically in the autotetraploid cytotype (50% lower mean correlations at 1 kb distance), suggesting that tight introgression signals may be formed rapidly in this system.

Although sharing of adaptive alleles between tetraploid populations can also be explained by ILS, the symmetrical design of our study allows us to reject ILS in most cases. Under ILS, we expect two divergent alleles to have existed in the ancestor of both species, which would lead to topology 6 after sorting of these alleles into diploids and tetraploids, respectively. Of the top 12 *Twisst* peaks, only one represents topology 6. The others represent topologies 11 and 14, in which the tetraploid alleles are nested within the diploids, implying that they arose after speciation and subsequently introgressed (Fig. 3a). A slight majority of these loci with introgression signal appear to have a *lyrata* origin (30/53 *Twisst* peaks), but among those with the highest levels of selection in *Lwt*, *Let*, and *arenosa*, a majority (11/16 where direction can be inferred) harbour evidence of an *arenosa* origin (Supplementary Dataset 4). GO enrichment analysis of the gene-coding loci in the windows where direction could be inferred found only enrichment for categories related to meiosis (Supplementary Datasets 5 and 6).

Taking these results together, we observe that four meiosis-related loci were outliers in all *Twisst* and divergence scans: *PRD3*, *ASY3*, *SYN1* and *DYAD*, and four did not show a signal in both *Twisst* analyses as well as both divergence scans: *ASY1*, *ZYP1a*, *ZYP1b* and *PDS5*. Given that *arenosa* is the much older tetraploid (2.5 times as ancient as *lyrata*; Fig. 1c) and is much more widespread, we hypothesise that *arenosa*-sourced alleles were under selection for stable polyploid meiosis longer, providing pre-adapted alleles to the nascent *lyrata* tetraploid, although this hypothesis needs to be functionally tested in dedicated studies. Introgression of optimised alleles from an older to a younger species has been indicated for high-altitude adaptive alleles from Denisovans and Tibetan *Homo sapiens*^42^. Taking *PRD3* as a clear example (Fig. 2b), derived *arenosa*-specific missense polymorphisms in the diploid population SNO are driven to high frequency in the tetraploids of both species, whereas diploid *lyrata* alleles are absent, strongly implicating a specific diploid *arenosa* origin in this case. At the same time, we detect specific signals of gene flow from *lyrata* into *arenosa*. In addition to meiosis-related genes, we see introgression signal at the endopolyploidy gene *CYCA2;3* and the global transcriptional regulator *TFIIF*, but very few other loci exhibit both persistent signatures of extreme selection as well as introgression (Supplementary Datasets 3 and 4).

Our findings suggest that introgression of particular alleles of meiosis-related genes might stabilise polyploid meiosis, with the less effective alleles of one species being replaced by introgression of alleles from orthologous loci in the other tetraploid. Introgression of alleles optimised for adaptation to WGD could be especially beneficial in hybrid zones such as this one, which spans a climatic gradient from a warmer, Pannonian climate at its eastern margin to harsher conditions in the eastern Alps. Meiosis is a temperature-sensitive process^15^ and we hypothesise substantial levels of meiosis-related allele–environment associations with variable temperature. Allele–environment associations with climatic variables across a hybrid zone have been observed in spruce^43^.

## Discussion

For these newly formed tetraploids, WGD appears to be both a blessing and a curse. Although WGD appears to have opened up access to the allelic diversity of a sister species, as well as provided population genomic benefits^21^, it also presents new challenges to the establishment of optimal allelic combinations. As the gene products at the meiotic loci under the most extreme selection across this hybrid zone functionally and physically interact, we expect that efficient evolved polyploid meiosis requires the harmonious interactions of multiple selected, introgressed alleles in concert. However, relatively high levels of residual masking of genetic load in autotetraploids^21,44^ will tend to extend the duration that deleterious alleles segregate in populations, with negative phenotypic consequences. This is consistent with our observation that polyploid meiosis exhibits wide degrees of within-population variability in stability. This observed diversity suggests that the optimal combination of meiosis alleles is yet segregating, which may also be the result of the recent age of these WGDs. Dedicated molecular investigation of whether the measured within-population meiotic stability is associated with particular allele combinations is the focus of ongoing functional analyses.

In this study, we investigated the population genetic basis of adaptation to WGD in congeners that, due to an endosperm-based postzygotic barrier^20^, hybridise only as tetraploids. We found that many of the same loci exhibit the most conspicuous signatures of selective sweep in *lyrata* following WGD that we observed in *arenosa*, and further, that the strongest signals of interspecific introgression occur precisely at many of these same loci. Using whole-genome sequence data from 30 populations, we probed complex population structure and patterns of gene flow. Interestingly, we observed cytologically that the degree of meiotic stability varied dramatically, even within populations of both species, suggesting that stability has not been completely established, or that other, perhaps epigenetic or environmental factors influence meiotic stability in still unknown ways. At the same time, populations exhibited admixture signals that contrast dramatically in degree, indicating a complex introgression landscape. We present evidence that the molecular basis by which WGD was stabilised in *lyrata* and *arenosa* is shared. Our data further suggest that WGD-facilitated hybridisation allowed for stabilisation of meiosis in nascent autotetraploids by specific, bidirectional adaptive gene flow, tightly overlapping loci known to be essential for processes that are impacted by WGD: meiotic stability, endopolyploidy, and transcription, and others. It is curious that the very process that rescues fitness in these species, hybridisation, is potentiated by the same phenomenon to which the resultant adaptive gene flow responds: WGD.

## Methods

### Sample design and sequencing

Individual plants were collected from field sites across Central Europe (Supplementary Fig. 1). Cytotypes were determined by flow cytometry from these populations in ref. ^8^ and ref. ^21^, no triploids have been detected in these populations, nor have we found any evidence in the flow cytometry or cytology data that any of these populations consist of mixed-ploidy subpopulations.

Central European tetraploid *lyrata* has its largest distribution in eastern Austria, in two biogeographic regions: the Wienerwald (*lyrata* eastern tetraploids/*Let* hereafter; LIC, MOD), and the Wachau (*lyrata* Wachau tetraploids/*Lwt* hereafter; PIL, SCB, KAG, SWA, LOI, MAU). We found an additional tetraploid *lyrata* population in Hungary (GYE) and included it in this study. Diploid *lyrata* populations were chosen from the Wienerwald (PEQ, PER, VLH), which are the geographically closest diploid populations to the *Let* and *Lwt*, and therefore likely serve as source populations.

For *arenosa*, representative populations of tetraploids from the Hercynian (WEK, SEN, BRD) and Alpine lineages (HOC, GUL, BGS) were selected, as well as additional *arenosa* populations from the Western Carpathians (diploid: SNO; tetraploid: TRE), which is the centre of *arenosa* genetic diversity and the region of origin of the tetraploid cytotype^9^. For breadth, we selected several more diploid *arenosa* populations from the Pannonian (KZL, SZI) and Dinaric (BEL) lineages, as well as the following populations from the hybrid zone in the eastern Austrian Forealps: HAL, ROK, FRE, OCH and KEH. To complement our sampling with diploid *lyrata* from across its entire distribution range, we selected samples from the Hercynian (SRR2040791, SRR2040804), arctic-Eurasian (SRR2040796, SRR2040798, SRR2040805) and arctic-North American lineages (DRR054584, SRR2040769, SRR2040770, SRR2040789). *A. croatica* (CRO) and *A. halleri* (SRR2040780, SRR2040782, SRR2040783, SRR2040784, SRR2040785, SRR2040786, SRR2040787) were included as outgroups^32^. The majority of *lyrata* and hybrid samples were collected as seeds, cultivated and flash-frozen prior to DNA extraction, whereas samples for three populations (LIC, MOD, HAL) were collected and silica-dried. Silica-dried material from GYE was obtained from Marek Šlenker and Karol Marhold. *Arenosa* samples were collected and sequenced as part of a different study^21^. In addition, 16 accessions were downloaded from the NCBI Sequence Read Archive (SRA), bringing the total sample number to 92 (Supplementary Table 1). DNA of the *lyrata* and hybrid samples was extracted and purified from frozen or silica-dried leaf and/or flower tissue using the Epicentre MasterPure DNA extraction kit. DNA concentration measurements were performed with the Qubit 3.0 fluorometer (Invitrogen/Life Technologies, Carlsbad, California, USA). Genomic libraries for sequencing were prepared using the Illumina TRUSeq PCR-free library kit with 500 ng to 1 μg extracted DNA as input. We multiplexed libraries based on the Qubit concentrations, and those multiplexed mixes were run on an initial quantification lane. According to the yields for each sample, loading of the same multiplex mix on several lanes was increased to achieve a minimum of 15× coverage. Samples that had less than our target coverage were remixed and run on additional lanes. Libraries were sequenced as 125 bp paired-end reads on a HiSeq2000 by the Harvard University Bauer Core Facility (Cambridge, MA, USA).

### Data preparation and genotyping

Newly generated sequencing data and SRA accessions were processed together from raw fastq reads. We first used Cutadapt^45^ to identify and remove adapter sequences with a minimum read length of 25 bp and a maximum error rate of 0.15. We then quality trimmed reads using TRIMMOMATIC^46^ (LEADING:10 TRAILING:10 SLIDINGWINDOW:4:15 MINLEN:50). Samples sequenced on several lanes were then concatenated using custom scripts. Reads were deduplicated using MarkDuplicates in picard v.1.103. Broadinst and readgroup names were adjusted utilising AddorReplaceReadGroups within the picard package. Reads were then mapped to the North American *lyrata* reference genome (v.2^47^) using bwa-mem in the default paired-end mode^48^. Indels were realigned using the Genome Analysis Toolkit (GATK) IndelRealigner. Prior to variant discovery, we excluded individuals that had fewer than 40% of bases <8× coverage (assessed via GATK’s DepthOfCoverage with the restriction to a minimum base quality of 25 and a minimum mapping quality of 25). Our final dataset for analysis contained 92 individuals.

Variant calling was performed using the GATK HaplotypeCaller (--min_base_quality_score 25 --min_mapping_quality_score 25 -rf DuplicateRead -rf BadMate -rf BadCigar -ERC BP_RESOLUTION -variant_index_type LINEAR -variant_index_parameter 128000 --pcr_indel_model NONE), followed by GenotypeGVCFs for genotyping. For each BAM file, HaplotypeCaller was run in parallel for each scaffold with ploidy specified accordingly and retaining all sites (variant and non-variant). We combined the single-sample GVCF output from HaplotypeCaller to multisample GVCFs and then ran GenotypeGVCFs to jointly genotype these GVCFs, which greatly aids in distinguishing rare variants from sequencing errors. Using GATK’s SelectVariants, we first excluded all indel and mixed sites and restricted the remaining variant sites to be biallelic. Additional quality filtering was performed using the GATK VariantFiltration tool (QD < 2, MQ < 40.00, FS > 60.0, SOR > 4.0, MQRankSum < − 8.0, ReadPosRankSum < − 8.0, DP < 8). Then we masked sites that had excess read depth, which we defined as 1.6× the second mode (with the first mode being heterozygous deletions or mismapping) of the read depth distribution.

### Population structure

All analyses dedicated to reveal population structure and demography were based on putatively neutral fourfold degenerate (4dg) single-nucleotide polymorphisms (SNPs) only. We used the 4dg filter generated for *arenosa* from ref. ^9^. After quality filtering, these analyses were based on a genome-wide dataset consisting of 4,380,806 4dg SNPs, allowing for a maximum of 10% missing alleles per site (1.2% missing data) at a 5× coverage minimum for a given individual sample.

Although we expected fastSTRUCTURE^49^ to be superior in recognising admixture compared with STRUCTURE^50^, running fastSTRUCTURE on our dataset resulted in poor performance, in that the result did not coincide with the STRUCTURE results or other analysis. This misbehaviour was probably due to the inclusion of polyploid data, as fastSTRUCTURE does not accommodate polyploid genotypes. We had randomly subsampled two alleles per each tetraploid site, similar to ref. ^32^, using a custom script. However, evidently such a subsampling strategy dissolves the fine-scale differences in admixture between populations at this scale. Hence, STRUCTURE was preferred, and was run on all samples and both ploidies. As STRUCTURE accepts only uniform ploidy as input, with one row per each ploidy, we added two rows of missing data for our diploid samples, making them pseudo-tetraploid. In addition, input data were LD-pruned and singletons removed using custom scripts. Window size was set to 500 with a distance of 1000 between windows, allowing for 10% missing data, which resulted in a dataset of 32,256 SNPs genome wide. We performed ten pruning replicates using the admixture model with uncorrelated allele frequencies, and then ran each for *K*-values 2–10 with a burn-in period of 50,000 and 500,000 Markov Chain Monte Carlo (MCMC) replicates. We conducted PCA using the glPca function in the adegenet *R* package^51^.

### Demographic parameters and reconstruction of gene flow

We next performed demographic analyses with *fastsimcoal2*^23^ on 4dg sites. A minimum of two individuals per each population was required. Custom python scripts (FSC2input.py at https://github.com/pmonnahan/ScanTools/) were used to obtain the multi-dimensional allele frequency (DSFS) spectrum as well as bootstrap replicates of the DSFS for confidence interval estimation. For the bootstrap replicates, the genome was divided into 50 kb segments and segments were resampled with replacement until recreating a DSFS of equivalent size as the genome. Ultimately, we aimed to estimate demographic parameters and confidence intervals for a four-population tree corresponding to diploid and tetraploid *lyrata* and *arenosa*. For computational efficiency, three-population trees were initially used to establish the presence/absence of migration edges by comparing models with a single migration edge to a null model with no migration. Additional migration edges would then be added and compared with the initial simple model. For each model, 50 replicates were performed and values kept for the replicate with the highest likelihood. For each replicate, we allowed for 40 optimisation cycles and 100,000 simulations in each step of each cycle for estimation of the expected side frequency spectrum. Although the above process identified the key migration edges, it resulted in a four-population tree that was overly complex; the exercise suggested six migration edges in total (Supplementary Fig. 3). Overfitting was evidenced by highly imprecise and nonsensical estimates for a subset of parameters (Supplementary Table 2). For example, the ancestral population size for *lyrata* was estimated to be greater than 5 million with individual replicate estimates ranging from <100,000 to over 10 million. Estimates for population fusion times were also drastically greater than observed in previous three-population trees. We therefore opted for a simpler model, retaining only the two migration edges with the highest support: bidirectional migration between tetraploids. Parameter estimates for each of the 100 bootstrap replicates were obtained using the scheme described above, and 95% confidence intervals were calculated using the 2.5th and 97.5th percentiles of the resulting distribution of each parameter.

### Changes in effective population size over time

PSMC model v.0.6.4 was used to infer changes in effective population size (*Ne*) through time using information from whole-genome sequences of *lyrata* and *arenosa* diploids^27^. We generated plots of the most deeply sequenced representative of each of the diploid *lyrata* and *arenosa* populations, with the exception of distinct *arenosa* KZL and SZI. A consensus fastq sequence was created using samtools v.1.2 and bcftools v.1.2 using samtools mpileup -C50 -Q 30 -q 30 with the *lyrata* v.2 genome as the reference. The reference was masked at all sites at which read depth was more than twice the average read depth across the genome. Samtools mpileup was followed with bcftools call -c and vcfutils.pl vcf2fq -d 5 -D 34 -Q 30 to create a fastq reference file. Using PSMC, this was changed to a format that was required with PSMC by fq2psmcfa -q20, and psmc was run with parameters psmc -N25 -t15 -r5 -p “4 + 25*2 + 4 + 6” and psmc_plot.pl -R -g 2 -u 3.7e-8 to get a text file that could be plotted with *R*. We used the mutation rate estimate *μ* = 3.7 × 10^−8^ ^9^ and a generation time of 2 years for both species, as *arenosa* is mainly biennial, and we estimate that *lyrata* generates the highest number of propagules in its second year after germination (R.S., personal observation).

### Cytological assessment of meiotic stability

Individual tetraploid *lyrata* and *arenosa* plants were germinated in 7 cm pots with Levington® Advance Seed and Modular Compost Plus Sand soil with 16 h light/8 h dark cycles at 20 °C constant temperature. Once rosettes had formed, plants were vernalised for six weeks with 8 h light (6 °C)/16 h dark (4 °C) cycles. Plants were then grown in 16 h light (13 °C)/8 h dark (6 °C) cycles to encourage flowering. Buds were collected, fixed and anthers dissected for basic cytology as described in^52^ except that 50 mg (30 Gelatine Digestive Units) Zygest® Bromelain were added to the enzyme mixture, and incubation time was increased to 75 min. The prepared slides were stained and mounted with 7 µl 4′,6-diamidino-2-phenylindole (DAPI) in Vectashield (Vector Lab) and metaphase I chromosomes visualised using a Nikon 90i Eclipse fluorescent microscope with NIS Elements software. Chromosome spreads with all rod and/or ring bivalents were scored as stable meiosis (Fig. 1d), whereas multivalents with multiple chiasmata were scored as unstable meiosis (Fig. 1e). FISH was performed as in^52^, except 62 °C was used as the chromosome denaturing temperature. The 5S rDNA probe was generated by directly incorporating biotin into a PCR product (Jenna Biosciences) using primers 5SF 5′-AACCGAAATTGCGTGCATAG-3′ and 5SR 5′-AAACGGGAGGTGAGACGAG-3′ with *Mimulus guttatus* cloned genomic DNA that shares 96% nucleotide identity with *A. lyrata* in this region and the 45S pTa71 clone (Gerlach and Bedbrook, 1979) by nick translation with digoxigenin (Jenna Biosciences). Streptavidin Dylight 594 and anti-digoxigenin Dylight 488 (Vector laboratories) were used as secondary fluorophores. Chromosomes were stained with DAPI in Vectashield (Vector Laboratories).

### Differentiation scans for signatures of selective sweeps

We grouped populations by ploidy level, species or hybrid affiliation, and affiliation to a biogeographic region in case of tetraploid *lyrata*. We calculated the following metrics in adjacent nonoverlapping genomic windows: AFD, *d*_*XY*_, Fst^53^, Rho^30^ and the number of fixed differences between the *lyrata* diploids and the two *lyrata* tetraploid groups (*Let* and *Lwt*). We identified selective sweep candidates as the 1% outliers of the empirical distribution for each metric. To maximise our chances of capturing differentiation truly related to ploidy and not local adaptation, we selected the overlap between these two independent scans wherein the tetraploids contrast by edaphic (soil) preference and then focused on outliers that were identified in a highly stringent genome scanning approach in *arenosa*^13^.

To obtain insight into differentiation between population groups, AFD, *d*_*XY*_, Fst, Rho, and the number of fixed differences were calculated for additional populations. *Arenosa* populations were grouped by lineage, as identified in refs. ^9,21^, as *arenosa* Hercynian tetraploids (*Aht*) and *arenosa* Alpine tetraploids (*Aat*), which also corresponds to biogeographic groupings.

### GO enrichment analysis

We performed gene function enrichment tests for each contrast using the CLUEGO app version 2.5.4 in CYTOSCAPE version 3.7.2 using GO information associated with orthologous *A. thaliana* gene identifiers. We retained levels 3-8 for biological process (Benjamini-Hochberg correction *p* ≤ 0.05).

### Visualisation of allele frequencies

We visualised allele frequencies of amino acid substitutions in form of a heatmap. Pre-processed VCF files were annotated using SnpEff^54^ (10.4161%2Ff) with the manually added *lyrata* v.2 reference annotation^55^ (10.1371/journa). Variants annotated as missense (i.e. amino acid substitutions) were extracted using SnpSift^54^. Gene-coding loci were extracted from the whole-genome annotated VCF and per-population allele frequencies for each amino acid substitution calculated using GATK’s SelectVariants. Alternative allele frequencies (polarised against the *lyrata* reference) were visualised using the heatmap.2 function in the gplots package in *R* (Warnes et al., 2016, https://CRAN.R-project.org/pac).

### Identification of differentiated and introgressed regions

To investigate how the relationships among diploid and tetraploid populations of the two species vary across the genome, we used topology weighting by iterative sampling of subtrees (*Twisst*)^40^ [www.github.com/simonhmartin/twisst/]. *Twisst* provides a quantitative measure of the relationships among a set of taxa when each taxon is represented by multiple individuals and the taxa are not necessarily reciprocally monophyletic. This provides a naive means to detect both introgression and ILS, and how these vary across the genome. We first inferred genealogies for 50 SNP windows across the whole genome using the BIONJ algorithm^56^ as implemented in PHYML^57^. As each individual carries two (for diploids) or four (for tetraploids) distinct haplotypes that represent different tips in the genealogy, it is necessary to first separate the haplotypes by phasing heterozygous genotypes. We used a heuristic approach to estimate phase that maximises the average extent of LD among all pairs of polymorphic sites in the window. This approach iteratively selects the best genotype configuration for each site, beginning with the site that has the most heterozygous genotypes. At each step, the optimal configuration is that which maximises the average LD between the target site and all previous target sites. This allows simultaneous phasing of diploids and tetraploids. We investigated the accuracy of this phasing approach using simulated sequences generated using the coalescent simulator *msms*^58^ and *seq-gen*^59^, following^40^, but here adding steps to randomise phase and then apply phase inference. As *Twisst* is robust to within-taxon phasing errors^40^, the relevant question here is the extent to which imperfect phasing would affect the estimated topology weightings. We therefore applied *Twisst* to the simulated data and compared the results with (i) perfect phase, (ii) randomised phase and (iii) randomised and then inferred phase. This confirmed that our heuristic phasing algorithm led to an improvement in the accuracy of the weightings, giving results that approached what is achieved with perfect phase information.

For running *Twisst* on the empirical data, we combined samples into four ingroup populations: diploid *lyrata*, tetraploid *lyrata*, diploid *arenosa* and tetraploid *arenosa*, and included *A. halleri* as outgroup. These five taxa give fifteen possible rooted taxon topologies (Fig. 3a). Although *Twisst* does not consider rooting when computing topology weightings, the inclusion of an outgroup improves the interpretation of the results, allowing the direction of introgression to be inferred in some cases^40^. In all analyses, topology weightings were computed exactly for all window trees that could be simplified to ≤2,000 remaining haplotype combinations (see ref. ^40^ for details). In cases where this was not possible, approximate weightings were computed by randomly sampling combinations of haplotypes until the 95% binomial confidence interval for all fifteen topology weightings was below 0.05. Confidence intervals were computed using the Wilson method implemented in the package binom in *R* (R Core Team 2015).

### Reporting summary

Further information on research design is available in the Nature Research Reporting Summary linked to this article.

## Supplementary information

Supplementary Information

Peer Review

Reporting Summary

Description of Additional Supplementary Files

Supplementary Dataset 1

Supplementary Dataset 2

Supplementary Dataset 3

Supplementary Dataset 4

Supplementary Dataset 5

Supplementary Dataset 6

Source Data

## Data Availability

Data supporting the findings of this work are available within the paper and its Supplementary Information files. A reporting summary for this Article is available as a Supplementary Information file. The datasets generated and analysed during the current study are available from the corresponding author upon request. All sequence data are freely available in the European Nucleotide Archive through accession code PRJEB34247. The source data underlying Figs. 1D, 1E, and 3 are provided as a Source Data file.

## Code availability

Custom programmes and scripts used in this study are available at GitHub: https://github.com/pmonnahan/ScanTools/ and https://github.com/simonhmartin/twisst/.
