# Supplementary material for: Interspecific introgression mediates adaptation to whole genome duplication
Source: Nat Commun. 2019 Nov 18;10:5218. doi: 10.1038/s41467-019-13159-5 (PMC6861236; doi:10.1038/s41467-019-13159-5)
Supplement: Supplementary file 1 — Supplementary Information [file 41467_2019_13159_MOESM1_ESM.pdf]

Interspecific introgression mediates adaptation to whole genome duplication

Marburger *et al.*

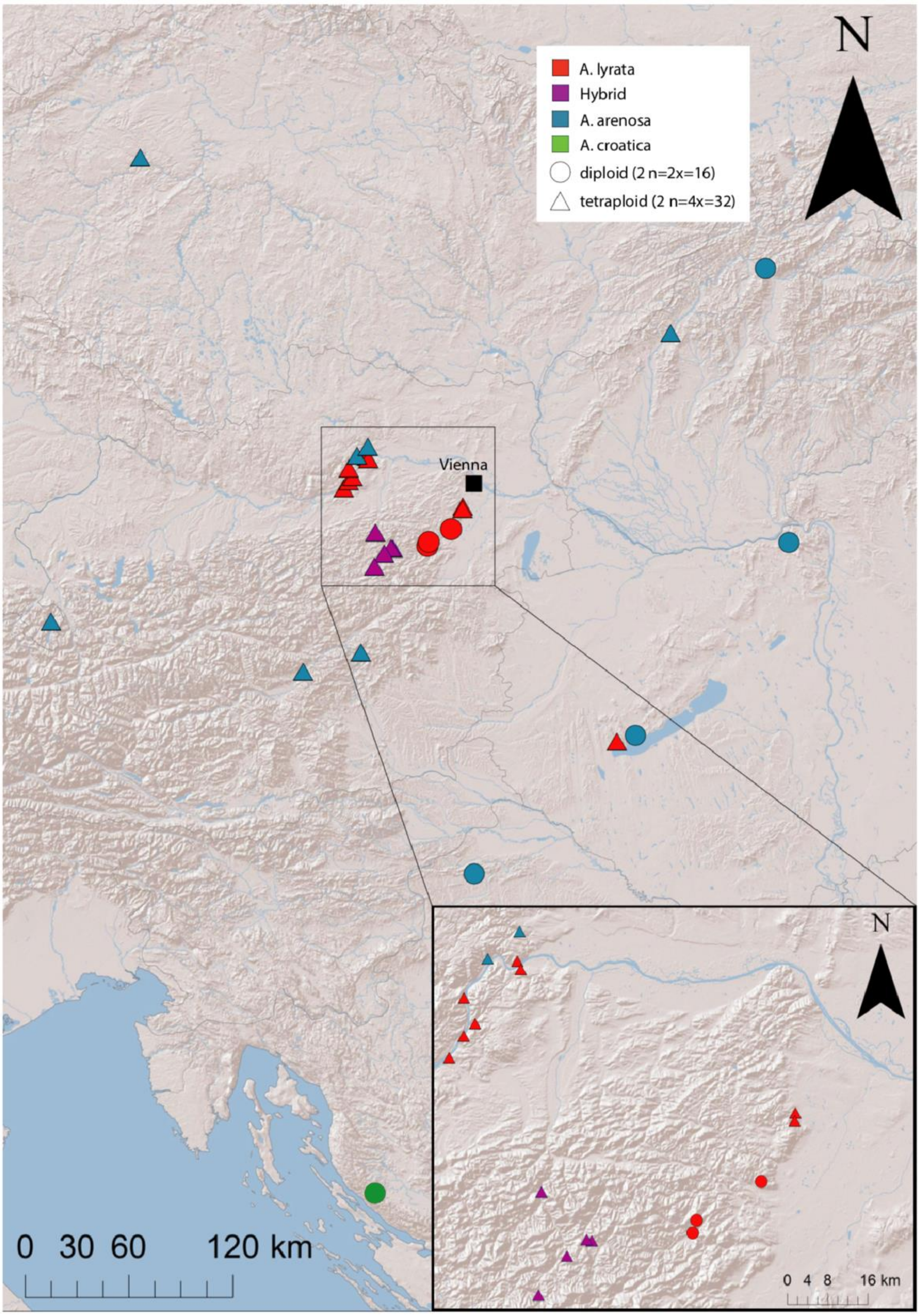

Supplementary Figure 1. Map of Central Europe showing the locations for populations sampled in this study and from Monnahan *et al.*[1]. Circles represent diploid populations, triangles represent tetraploid populations. Colours are indicative of species and hybrids as indicated in the inset. The inset represents a zoomed in view of the eastern Austrian Forealps and the Wachau valley.

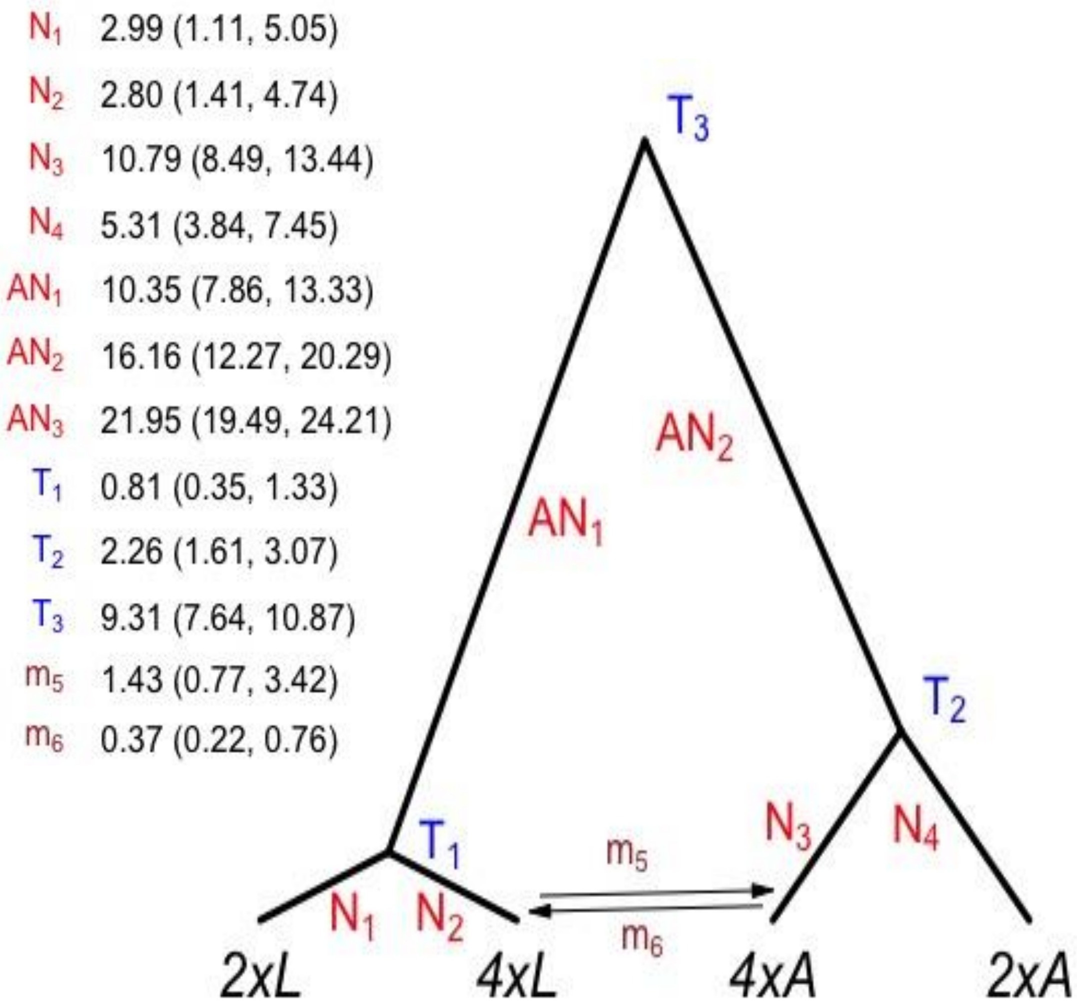

Supplementary Figure 2. Demographic parameter estimates. Units are 100,000's of individuals for population size (N and AN), 100,000's of generations for time estimates (T), and 10^{-6} alleles per generation (m). Median values across replicates are given. Note that gene flow estimates (m_5 and m_6) must be multiplied by Ne for alleles/generation per haploid genome (and this value therefore divided by the ploidy). *2xL* refers to diploid *A. lyrata*, *4xL* refers to tetraploid *A. lyrata*, *4xA* refers to tetraploid *A. arenosa* and *2xA* refers to diploid *A. arenosa*.

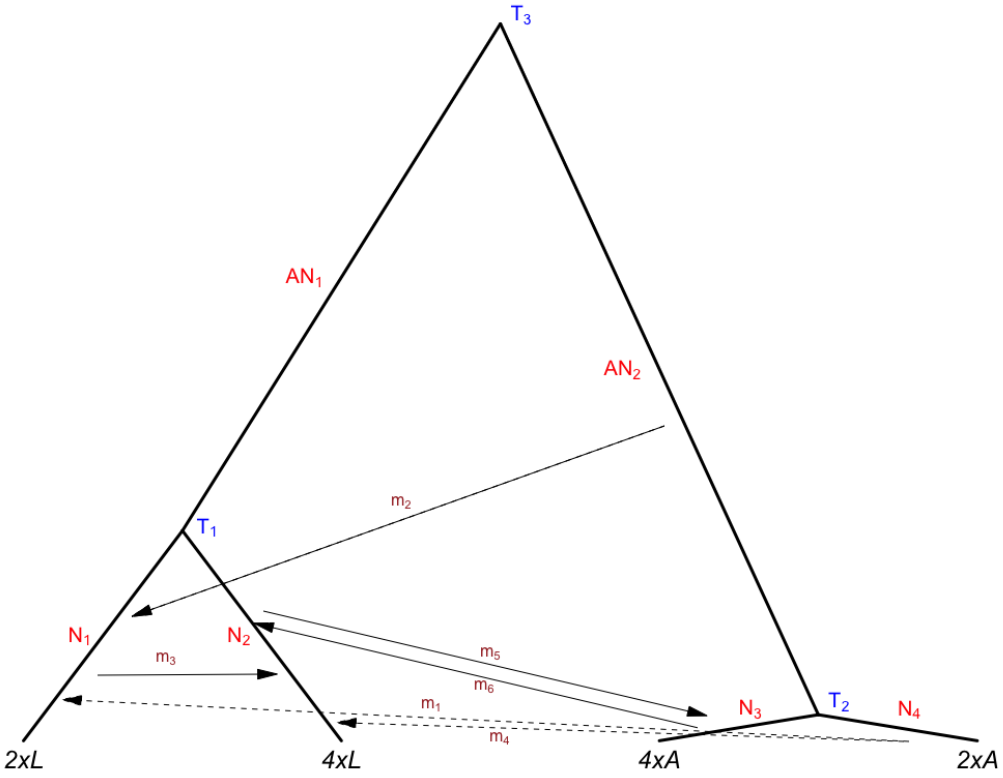

Supplementary Figure 3. Parameter estimates for the different 4 population scenarios. Parameter values are given in Supplementary Table 2.

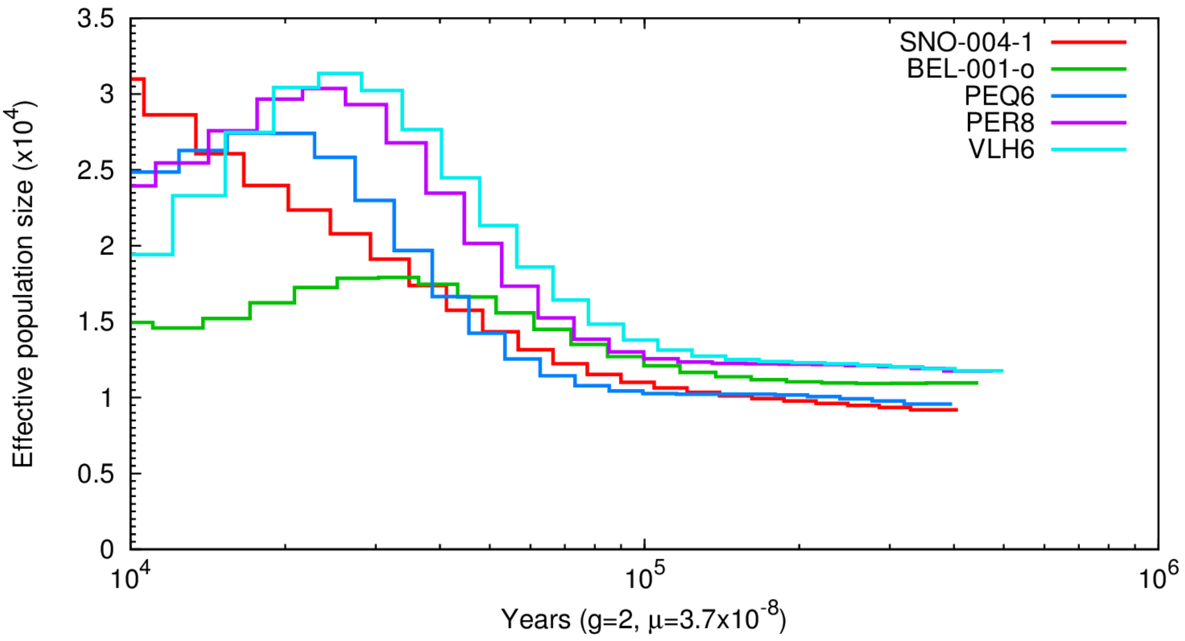

Supplementary Figure 4. Pairwise Sequentially Markovian Coalescent Model analysis of diploid *A. lyrata* and *A. arenosa* populations used in this study. We used the mutation rate $\mu=3.7\text{x}10^{-8}$ [2] and a generation time of two years for both species, as *A. arenosa* is mainly biennial, and we suppose that *A. lyrata* generates the highest number of propagules in its second year after germination.

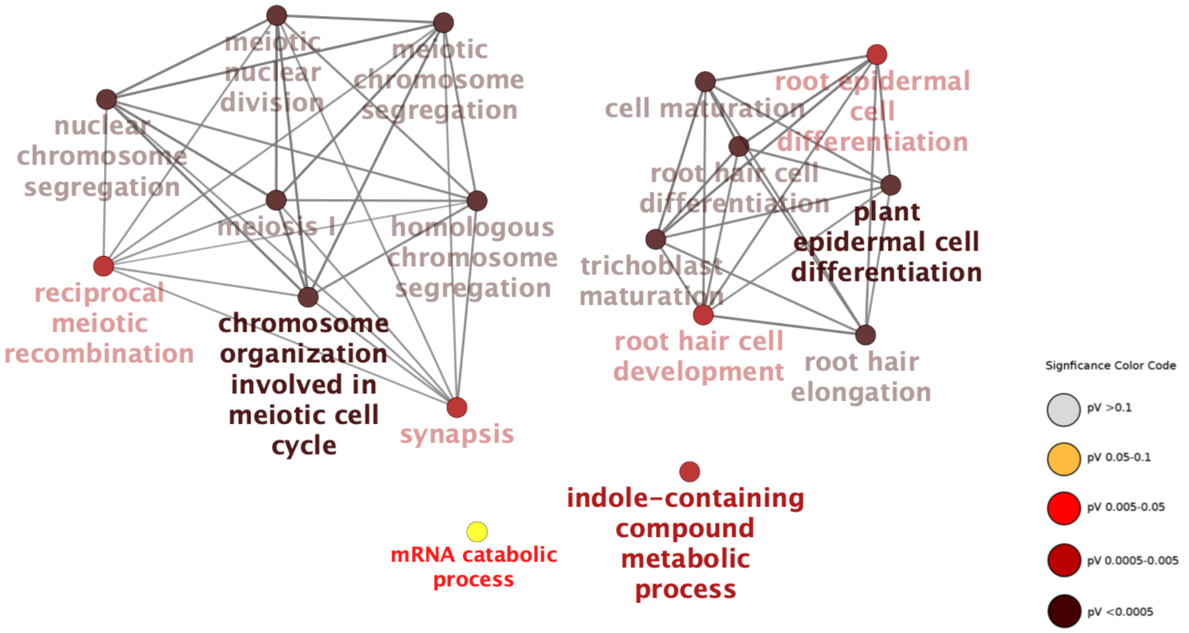

Supplementary Figure 5. Significant gene ontology enrichment for diverse polyploidy-related functional categories in *Arabidopsis lyrata*. Functional enrichment among 196 gene coding loci comprising the *Let/Lwt* overlap list of outliers (Benjamini-Hochberg correction $p \leq 0.05$).

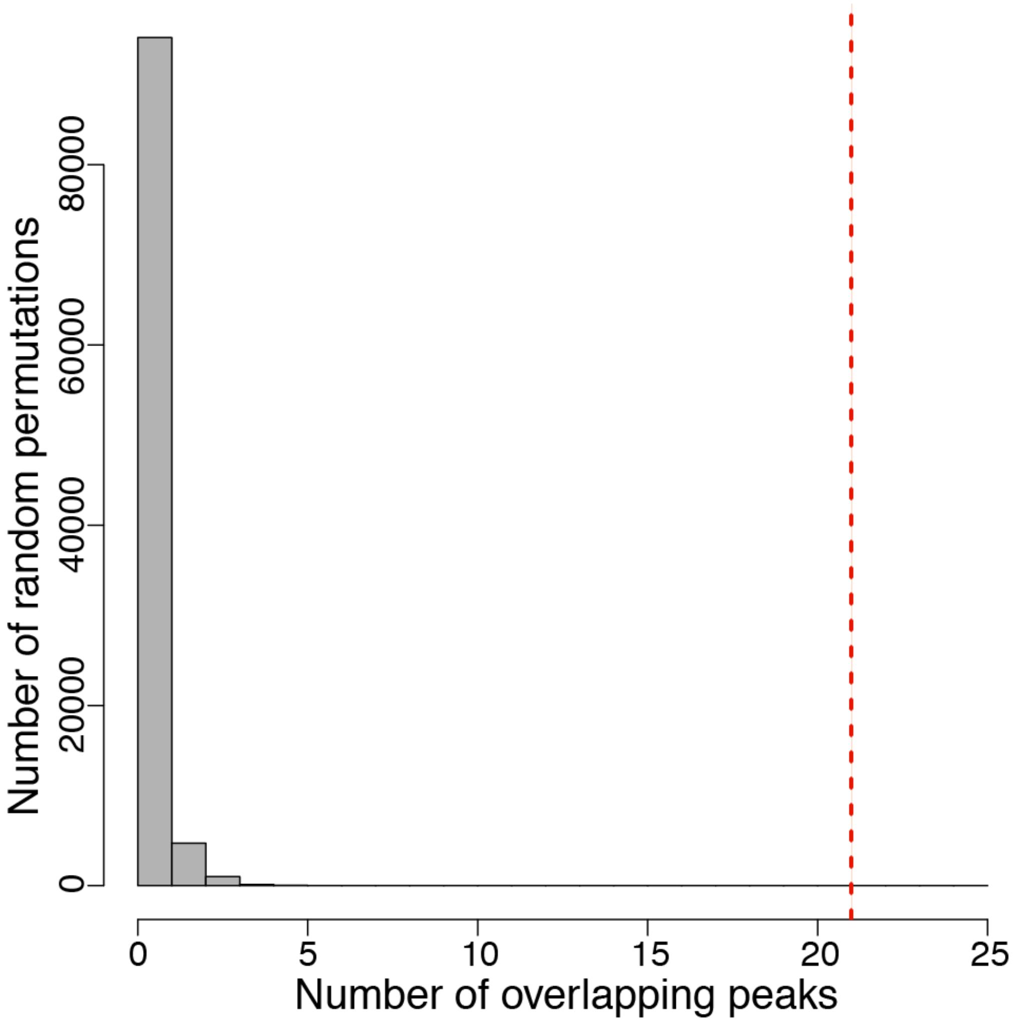

Supplementary Figure 6. Permutation tests indicating expected distribution of degree of overlap observed between *Twisst* outlier windows and divergence scan outlier windows. Red line indicates the observed value of 21 overlapping gene coding loci exhibiting both *Twisst* outlier and divergence scan outlier status, many more than expected by chance.

Supplementary Table 1. The 30 populations included in this study.

Pop.	N ind.	Ploidy	Altitude	Latitude	Longitude	Country	Locality	Published in (orig. code)	Origin of tissue
BEL	3	2x	550	46.16167	16.11500	HR	Castle ruin Belecgrad; open sites in the forest, walls of the castle ruin	Monnahan *et al.*[1] (BEL)	wild
BGS	3	4x	570	47.62806	13.00167	D	Berchtesgaden; railway, secondary gravel	Hollister *et al.*[3] (BGS)	cultiv.
BRD	2	4x	350	50.04967	13.89081	CZ	Brdatka; open forest in canyon of Berounka river	Monnahan *et al.*[1] (BRD)	wild
CRO	4	2x	1076	44.53147	15.19402	HR	Ljubičko Brdo and Zavižan; rocks	Monnahan *et al.*[1] (CRO)	wild
FRE	2	4x	391	47.99405	15.57118	AT	Freiland; rocks		cultiv.
GUL	3	4x	820	47.29000	14.93167	AT	Gulsen; serpentine rocks	Arnold *et al.*[4] (GU)	cultiv.
GYE	3	4x	202	46.78442	17.28775	HU	Gyenesdiás; dolomitic rock on northern border of the village		wild
HAL	3	4x	665	47.90187	15.69233	AT	Halbach valley; gravel		wild
HOC	3	4x	580	47.37000	15.38667	AT	Hochlantsch	Arnold *et al.*[4] (HO)	cultiv.
KAG	3	4x	257	48.29432	15.42614	AT	Wachau, Kartause Aggsbach; rocks		cultiv.
KEH	2	4x	699	47.81611	15.54311	AT	Kernhof; rocks, gravel		cultiv.
KZL	3	2x	330	47.72444	18.77917	HU	Kesztölc	Monnahan *et al.*[1] (KZL)	cultiv.
LIC	3	4x	298	48.09283	16.27073	AT	Liechtenstein castle; walls of the castle ruin, rocks		wild
LOI	3	4x	289	48.39649	15.55268	AT	Wachau, Loibenberg; oak-pine forest, rocks		cultiv.
MAU	3	4x	244	48.3818	15.56031	AT	Wachau, Mauternbach; oak-pine forest, rocks		cultiv.
MOD	3	4x	335	48.07955	16.26718	AT	Castle ruin Mödling; walls of the castle ruin, rocks		wild
OCH	2	4x	698	47.87950	15.62691	AT	Untermitterbach, road to Ochsattel; gravel		cultiv.
PEQ	2	2x	461	47.90154	15.96864	AT	Pernitz, small quarry in Haltergraben; rocks		cultiv.
PER	2	2x	564	47.92251	15.98176	AT	Pernitz, road from Pernitz to Pottenstein; rocks		cultiv.
PIL	3	4x	224	48.23901	15.34931	AT	Wachau, mouth of Pielach river into Danube river; rocks		cultiv.
ROK	2	4x	662	47.90531	15.68304	AT	Rossbachklamm; rocks, gravel		cultiv.
SCB	2	4x	244	48.27428	15.39301	AT	Wachau, Schönbühel; rocks		cultiv.
SEN	1	4x	296	48.44750	15.56469	AT	Castle ruin Senftenberg; walls of the castle ruin, rocks		cultiv.
SNO	2	2x	390	49.17417	18.86167	SK	Strečno	Yant *et al.*[5] (SN)	cultiv.
SWA	2	4x	264	48.34031	15.40085	AT	Wachau, Schwallenbach; rocks		cultiv.
SZI	3	2x	130	46.80667	17.43444	HU	Szigligeti vár	Monnahan *et al.*[1] (SZI)	cultiv.
TBG		4x	640	48.13972	8.23667	D	Triberg; railway, secondary gravel	Hollister *et al.*[3] (TBG)	cultiv.
TRE	3	4x	280	48.89417	18.04472	SK	Trenčín; rocks at the castle ruin	Monnahan *et al.*[1] (TRE)	cultiv.
VLH	3	2x	484	47.97978	16.16374	AT	Vöslauer Hütte; pine forest, rocks		cultiv.
WEK	3	4x	359	48.40502	15.47291	AT	Wachau, Weißenkirchen; oak-pine forest, former vineyard	Monnahan *et al.*[1] (WEK)	wild

Supplementary Table 2. Parameter estimates, likelihood, and AIC for different 4-population scenarios in *fastsimcoal2*.

Variable	Model1	Model2	Model3	Model4	Model5	Model6
N1	465938	482982	437237	303955	110886	369141
N2	75834	88151	188323	289967	373998	339673
N3	836187	873906	927130	1079527	1120090	1091553
N4	561181	461999	474882	540394	566028	473956
AN1	4959828	4376433	3004479	1036598	1162941	934617
AN2	2036501	2011382	1901016	1611485	1169502	1746457
AN3	2574717	2003004	2072445	2207793	2354880	2249547
T1	2034971	383815	253022	81326	47876	99582
T2	217193	182807	190528	230060	247875	210015
T3	6589497	1238849	1095980	930721	613422	838791
m1	2.79E-07	--	--	--	--	--
m2	7.48E-07	--	--	--		--
m3	1.71E-05	2.44E-05	8.28E-06	--	--	--
m4	1.18E-06	1.28E-06	--	--	--	--
m5	3.61E-07	5.80E-07	4.30E-07	4.29E-07	8.14E-07	--
m6	2.71E-06	4.34E-06	2.21E-06	1.57E-06	--	1.26E-06
Likelihood	-623798	-622710	-622579	-622342	-625995	-622483
AIC	2872730	2867798	2867139	2865432	2882840	2866662

Note: Model 4 with the highest likelihood and lowest AIC was chosen. Mean values are given, in contrast to medians in Figure 1C and Supplementary Figure 2, in order to get an estimate across replicates. Gene flow estimates must be multiplied by Ne for alleles/generation per haploid genome.

Supplementary Table 3. Chromosome stability scoring of individual plants from tetraploid populations of *A. lyrata* and *A. arenosa* and a hybrid population at meiotic metaphase I.

Population	Plant No.	No. cells with M1s scored	No. cells with stable M1s	No. cells with unstable M1s	% stable	% unstable
**LIC**	1	11	10	1	**91**	9
	2	11	3	8	**27**	73
	3	18	3	15	**17**	83
	4	54	35	19	**65**	35
	5	13	9	4	**69**	31
**MOD**	1	20	15	5	**75**	25
	2	63	51	12	**81**	19
	3	8	7	1	**88**	13
	4	21	1	20	**5**	95
**KAG**	1	24	23	1	**96**	4
	2	31	29	2	**94**	6
	3	27	0	27	**0**	100
	4	58	57	1	**98**	2
	5	36	28	8	**78**	22
**ROK**	1	15	3	12	**20**	80
	2	39	31	8	**79**	21
	3	26	15	11	**58**	42
	4	31	20	11	**65**	35
	5	39	32	7	**82**	18
**WEK**	1	61	56	5	**92**	8
	2	30	29	1	**97**	3
	3	17	17	0	**100**	0
	4	26	26	0	**100**	0
	5	35	30	5	**86**	14
**SEN**	1	46	43	3	**93**	7
	2	49	43	6	**88**	12
	3	37	35	2	**95**	5
	4	47	42	5	**89**	11
	5	42	37	5	**88**	12
**TBG**	1	53	40	13	**75**	25
	2	22	18	4	**82**	18
	3	52	45	7	**87**	13
	4	14	11	3	**79**	21
	5	74	70	4	**95**	5

Note: Chromosome spreads with all rod and/or ring bivalents were scored as “Stable meiosis” (Figure 1D), while multivalents with multiple chiasmata were scored as “Unstable meiosis” (Figure 1E). Tetraploid *A. lyrata*: LIC, MOD, KAG. Hybrid: ROK. Tetraploid *A. arenosa*: WEK, SEN, TBG. The TBG population was not integrated in the other parts of this study, but is included for comparison; it was the tetraploid *A. arenosa* population on which the study of [5] was based. MI refers to meiotic metaphase I.

Supplementary Table 4. Differentiation between various contrasts.

	**Contrast**	**No. SNPs**	**AFD**	d_{XY}	**Fst**	**Rho**	**Fixed Diff**
Lyrata diploid vs. tetraploid	*Lyd* vs. *Let*	2,904,110	0.14	0.22	0.09	0.19	270
	Lyd vs. *Lwt*	3,794,257	0.11	0.16	0.07	0.17	64
Lyrata tetraploid vs. tetraploid	*Let* vs. *Lwt*	4,795,381	0.09	0.16	0.06	0.13	24
Arenosa tetraploid vs. tetraploid	*Aht* vs. *Aat*	1,812,223	0.10	0.16	0.03	0.07	0
Lyrata vs. *arenosa*	*Lyd* vs. *Aht*	1,729,114	0.25	0.27	0.39	0.39	41,810
	Lyd vs. *Aat*	2,874,610	0.23	0.24	0.40	0.40	57,492
	Let vs. *Aht*	2,257,560	0.21	0.24	0.34	0.36	17,000
	Lwt vs. *Aht*	2,513,764	0.15	0.19	0.26	0.32	767
	Let vs. *Aat*	3,644,666	0.20	0.23	0.35	0.37	21,372
	Lwt vs. *Aat*	3,653,076	0.16	0.20	0.29	0.34	947
Hybrids from the eastern Austrian Forealps vs. *lyrata* or *arenosa*	*Hy1* vs. *Lyd*	4,165,349	0.16	0.20	0.17	0.29	259
	Hy2 vs. *Lyd*	4,055,168	0.16	0.20	0.15	0.27	322
	Hy1 vs. *Aat*	3,797,036	0.11	0.18	0.11	0.20	2
	Hy2 vs. *Aat*	3,752,996	0.13	0.19	0.15	0.24	43

Note: Genome-wide metrics of differentiation are allele frequency differences (AFD), d_{XY}, Fst, Rho, and the number of fixed differences (Fixed Diff). Diploid *A. lyrata*: *Lyd*. Tetraploid *A. lyrata*: *lyrata* eastern tetraploids (*Let*), *lyrata* Wachau tetraploids (*Lwt*). Tetraploid *A. arenosa*: *arenosa* Hercynian tetraploids (*Aht*), *arenosa* Alpine tetraploids (*Aat*). Hybrids from the eastern Austrian Forealps: HAL, ROK, FRE, OCH, KEH (*Hy1*), and, alternatively, HAL, ROK, FRE, OCH (*Hy2*); the distinction between *Hy1* and *Hy2* was made because only HAL, ROK, FRE, and OCH are intermediate hybrids (KEH is more *arenosa*-like).

Supplementary References

1. Monnahan, P. *et al.* Pervasive population genomic consequences of genome duplication in *Arabidopsis arenosa*. *Nat. Ecol. Evol.* **3**, 457-468 (2019).

2. Arnold, B., Kim, S. T. & Bomblies, K. Single geographic origin of a widespread autotetraploid *Arabidopsis arenosa* lineage followed by interploidy admixture. *Mol. Biol. Evol.* **32**, 1382-1395 (2015) doi:10.1093/molbev/msv089.

3. Hollister, J. D. *et al.* Genetic Adaptation Associated with Genome-Doubling in Autotetraploid *Arabidopsis arenosa*. *PLoS Genet.* **8** (2012) doi:10.1371/journal.pgen.1003093.

4. Arnold, B. J. *et al.* Borrowed alleles and convergence in serpentine adaptation. *Proc. Natl. Acad. Sci. U. S. A.* **113**, 8320-8325 (2016).

5. Yant, L. *et al.* Meiotic adaptation to genome duplication in *Arabidopsis arenosa*. *Curr. Biol.* **23**, 2151-2156 (2013).
