# Supplementary material for: Interspecific introgression mediates adaptation to whole genome duplication
Source: Nat Commun. 2019 Nov 18;10:5218. doi: 10.1038/s41467-019-13159-5 (PMC6861236; doi:10.1038/s41467-019-13159-5)
Supplement: Supplementary file 3 — Reporting Summary [file 41467_2019_13159_MOESM3_ESM.pdf]

nature research

Corresponding author(s): Levi Yant and Roswitha Schmickl

# Reporting Summary

Nature Research wishes to improve the reproducibility of the work that we publish. This form provides structure for consistency and transparency in reporting. For further information on Nature Research policies, see Authors & Referees and the Editorial Policy Checklist.

## Statistical parameters

When statistical analyses are reported, confirm that the following items are present in the relevant location (e.g. figure legend, table legend, main text, or Methods section).

| n/a | Confirmed | |
|---|---|---|
| ☐ | ☒ | The exact sample size ($n$) for each experimental group/condition, given as a discrete number and unit of measurement |
| ☐ | ☒ | An indication of whether measurements were taken from distinct samples or whether the same sample was measured repeatedly |
| ☐ | ☒ | The statistical test(s) used AND whether they are one- or two-sided<br>*Only common tests should be described solely by name; describe more complex techniques in the Methods section.* |
| ☐ | ☒ | A description of all covariates tested |
| ☐ | ☒ | A description of any assumptions or corrections, such as tests of normality and adjustment for multiple comparisons |
| ☐ | ☒ | A full description of the statistics including central tendency (e.g. means) or other basic estimates (e.g. regression coefficient) AND variation (e.g. standard deviation) or associated estimates of uncertainty (e.g. confidence intervals) |
| ☐ | ☒ | For null hypothesis testing, the test statistic (e.g. $F$, $t$, $r$) with confidence intervals, effect sizes, degrees of freedom and $P$ value noted<br>*Give P values as exact values whenever suitable.* |
| ☒ | ☐ | For Bayesian analysis, information on the choice of priors and Markov chain Monte Carlo settings |
| ☒ | ☐ | For hierarchical and complex designs, identification of the appropriate level for tests and full reporting of outcomes |
| ☐ | ☒ | Estimates of effect sizes (e.g. Cohen's $d$, Pearson's $r$), indicating how they were calculated |
| ☐ | ☒ | Clearly defined error bars<br>*State explicitly what error bars represent (e.g. SD, SE, CI)* |

*Our web collection on statistics for biologists may be useful.*

## Software and code

Policy information about availability of computer code

| | |
|---|---|
| Data collection | No software was used for data collection. |
| Data analysis | We used cutadapt 1.9, bwa 0.7.12, samtools 0.1.19, stampy 1.0.28, samtools 0.1.19, picard 1.134, GATK 3.5.0, GATK 3.6.0, fastsimcoal2 v.25 , adegenet , StAMPP , SplitsTree , RAxML v.8 , Astral v.4.10.10 , fastStructure v.1.0 , STRUCTURE v.2.3.2 , Treemix v.1.3 , pegas R package , ade4 R package , org.ASM (http://pythonhosted.org/ORG.asm), Mafft, PSMC 0.6.4 , https://github.com/pmonnahan/ScanTools, Twisst , MATLAB (MathWorks), Stampy , and the lme4 R package. |

For manuscripts utilizing custom algorithms or software that are central to the research but not yet described in published literature, software must be made available to editors/reviewers upon request. We strongly encourage code deposition in a community repository (e.g. GitHub). See the Nature Research guidelines for submitting code & software for further information.

## Data

Policy information about availability of data

All manuscripts must include a data availability statement. This statement should provide the following information, where applicable:

- Accession codes, unique identifiers, or web links for publicly available datasets
- A list of figures that have associated raw data
- A description of any restrictions on data availability

Sequence data that support the findings of this study have been deposited in the European Nucleotide Archive [ENA; https://www.ebi.ac.uk/ena] with the primary accession code PRJEB34247

# Field-specific reporting

Please select the best fit for your research. If you are not sure, read the appropriate sections before making your selection.

☐ Life sciences ☐ Behavioural & social sciences ☒ Ecological, evolutionary & environmental sciences

For a reference copy of the document with all sections, see nature.com/authors/policies/ReportingSummary-flat.pdf

# Ecological, evolutionary & environmental sciences study design

All studies must disclose on these points even when the disclosure is negative.

| | |
|---|---|
| Study description | We analysed the genomes of 291 individual Arabidopsis spp plants from 40 populations (including outgroups) and performed a population genomic analysis. We sampled between 5 and 12 individuals per population to estimate allele frequencies. |
| Research sample | Our primary focal populations were 39 populations (287 Arabidopsis arenosa plants) sampled from throughout their native range in Europe. A population is defined as a single group of plants separated from others by at least 500 meters. All samples were collected with appropriate permissions from relevant local and national authorities. All sampling was non-destructive, consisting of collection of several seeds from each plant or a sample of leaf materials, as described in the methods. |
| Sampling strategy | Sample sizes (numbers of genomes sequenced) were chosen to optimise the tradeoff between numbers of populations studied and alleles sampled in each population to obtain reliable estimates across genomes, based on previous work (Yant et al 2013 and Arnold et al 2016, cited in the main MS). |
| Data collection | We did not collect data from the wild; we analyzed sequence data. This was performed by most of the team as indicated by the author contributions statement. |
| Timing and spatial scale | Populations were collected between May 2012 and March of 2013 from across the natural range of A. arenosa in as even manner as we could manage given time and funding constraints. |
| Data exclusions | Following sequencing we excluded individuals from downstream analysis that had excessively low coverage, which would bias genome analysis: 'Prior to variant discovery, we excluded individuals that had less than 40% of bases <4× coverage (assessed via GATK 'DepthOfCoverage' with the restriction to a minimum base quality of 25 and a minimum mapping quality of 25).' This exclusion criterion was pre-established. |
| Reproducibility | We did not replicate the entire study but we replicated heavily during the demographic analyses (esp fastsimcoal2 coalescent modelling) by sampling different populations for each trio analysis and we report clearly the agreement of these replicated sampled analyses. |
| Randomization | We did not randomise populations sampled. We sampled according to known ecological, demographic, and genetic factors. |
| Blinding | Blinding was not possible in our genomic analysis. |

Did the study involve field work? ☐ Yes ☒ No

# Reporting for specific materials, systems and methods

## Materials & experimental systems

| n/a | Involved in the study |
|---|---|
| ☒ | ☐ Unique biological materials |
| ☒ | ☐ Antibodies |
| ☒ | ☐ Eukaryotic cell lines |
| ☒ | ☐ Palaeontology |
| ☒ | ☐ Animals and other organisms |
| ☒ | ☐ Human research participants |

## Methods

| n/a | Involved in the study |
|---|---|
| ☒ | ☐ ChIP-seq |
| ☒ | ☐ Flow cytometry |
| ☒ | ☐ MRI-based neuroimaging |
