# Supplementary material for: Interspecific introgression mediates adaptation to whole genome duplication
Source: Nat Commun. 2019 Nov 18;10:5218. doi: 10.1038/s41467-019-13159-5 (PMC6861236; doi:10.1038/s41467-019-13159-5)
Supplement: Supplementary file 4 — Description of Additional Supplementary Files [file 41467_2019_13159_MOESM4_ESM.docx]

**Description of Additional Supplementary Files**

File name: Supplementary Data 1

Description: Gene coding loci that repeatedly emerged as top 1% empirical outliers in two independent genome scans (diploid A. lyrata vs. Let populations [“Let scan”] and diploid A. lyrata vs. Lwt [“Lwt scan”]).

File name: Supplementary Data 2

Description: Results from GO enrichment analysis of Let Lwt overlap divergence outliers.

File name: Supplementary Data 3

Description: Results from GO enrichment analysis of list of genes that overlap all three scans: Let, Lwt and (Yant 2013 outliers)

File name: Supplementary Data 4

Description: Genomic windows with weightings above 0.5 for topologies 6, 11 and 14 in the Twisst analyses. Genes-coding loci found in both Twisst analyses are indicated in bold.

File name: Supplementary Data 5

Description: Results from GO enrichment analysis of list of genes that overlap Let Lwt divergence scan and Arenosa to Lyrata gene flow topology by Twisst analysis

File name: Supplementary Data 6

Description: Results from GO enrichment analysis of list of genes that overlap Let Lwt divergence scan and Lyrata to Arenosa gene flow topology by Twisst analysis
