# Supplementary figures and images for: Interspecific introgression mediates adaptation to whole genome duplication
Source: Nat Commun. 2019 Nov 18;10:5218. doi: 10.1038/s41467-019-13159-5 (PMC6861236; doi:10.1038/s41467-019-13159-5)

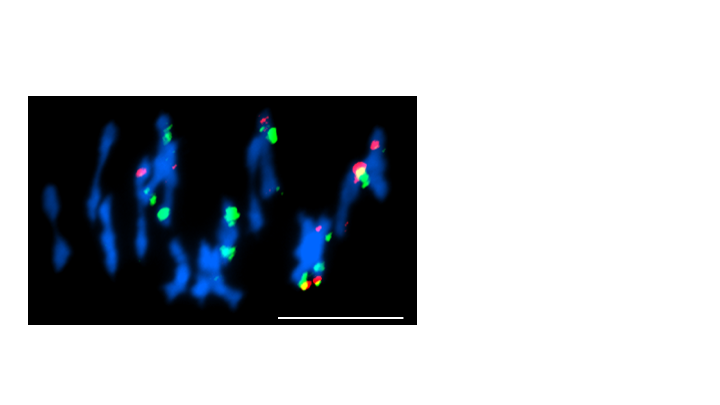

Supplement: Supplementary file 11 — Source Data [file 41467_2019_13159_MOESM11_ESM.zip › 1D.tiff]

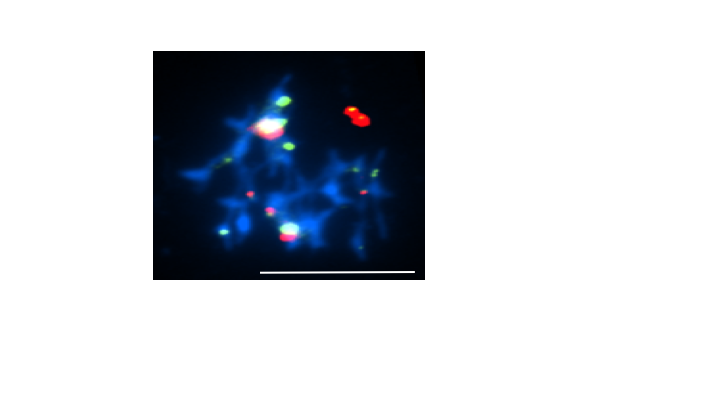

Supplement: Supplementary file 11 — Source Data [file 41467_2019_13159_MOESM11_ESM.zip › 1E.tiff]
