# Supplementary material for: Interspecific introgression mediates adaptation to whole genome duplication
Source: Nat Commun. 2019 Nov 18;10:5218. doi: 10.1038/s41467-019-13159-5 (PMC6861236; doi:10.1038/s41467-019-13159-5)
Supplement: Supplementary file 2 — Peer Review [file 41467_2019_13159_MOESM2_ESM.pdf]

Reviewer #1 (Remarks to the Author):

Review of 'Interspecific introgression mediates adaptation to whole genome duplication'

Marburger at el. describes a population genomic study in which they test the hypothesis that adaptive gene flow (of genes adapted to increase meiotic stability and/or reduce meiotic crossovers) from one species to another mediates escape from extinction following WGD. In particular, the authors show that specific pre-adapted alleles 'donated' from tetraploid Arabidopsis arenosa to tetraploid Arabidopsis lyrata (which has undergone a WGD more recently than arenosa) probably rescued the nascent A. lyrata tetraploids from early extinction.

I have read this paper with great interest, but have the following comments.

The authors mention several times that the pre-adapted alleles donated from A. arenosa to A. lyrata are responsible for rescuing A. lyrata from extinction. However, to this reviewer it is unclear whether tetraploid A. lyrata would indeed have gone extinct without adaptive introgression of alleles from A. arenosa or just adapt to WGD more slowly? Is there any way to 'proof' that, if not having obtained these adapted alleles by gene flow, the new tetraploids would have gone extinct? Is this suggested by the paragraph 'stabilisation of lyrata meiosis following WGD', where the authors mention that meiotic stability is segregating within tetraploid populations? This is not entirely clear to me. I have the feeling the authors might be more careful in phrasing this. I think the observation that pre-adapted genes have been obtained by gene flow is very interesting and for sure are a strong indication that these helped A. lyrata coping with the WGD (as has been shown previously for A. arenosa), but this is not the same as assuming that otherwise tetraploid A. lyrata should have gone extinct for sure. If true, then, we also need to assume that A. arenosa would have received its 'adapted' genes from somewhere else (or it too might have gone extinct ..).

The authors state that '… selective sweep signatures directly overlapping eight loci that interact during prophase I..'. I assume that it is not the genetic loci that directly interact but rather their gene products?

The authors state that they '… estimated the age of WGDs at 81k generations for lyrata (~160,000 years)'. In this respect, I think the following papers could be cited as well (Hohmann and Koch, 2017; Novikova et al., 2018).

The authors state that interspecific gene flow from tetraploid arenosa to tetraploid lyrata (0.0998 alleles/generation) and lyrata to arenosa are at the same level (0.1001 alleles/generation)"
How come arenosa->lyrata flow is lower than lyrata->arenosa? Admixture patterns and Dfoil results suggest the opposite, or am I missing something here?

The authors experimentally show that meiotic stability is varying from population to population (Table S3), but they fail to show if it depends on the presence of particular sets of adaptive alleles (from Table 2, for example) or admixture assignments. Is this something that could be looked at?

In general, I think it would be nice if the authors could briefly explain the population statistics they used (I, for example had to look up Rho, and there are several others like this for which I'm not sure the average reader will know what the authors mean with these).

The authors state that 'Overall, genome-wide differentiation between lyrata diploids and the tetraploid populations was extremely low (mean Rho between ploidies = 0.19)'. Low compared to what? It is the highest value in the table?! Table 1 needs the same statistics for diploid vs. tetraploid A. arenosa. I think this is what they compare it with?

Fig. 2A should be better explained. What is the red line? There are two y-axes, however, which one is

which (dots/line)?

'..tetraploids, Lwt hereafter: PIL, SCB, KAG, SWA, LOI, MAU). ' What happened to the GYE population? It is included in the STRUCTURE analysis but not in any of the scans. Or am I missing something?

Figure 3 shows clear signal for introgression from lyrata to arenosa (topo 11) as well, which doesn't overlap with genome scans and is probably not involved in WGD adaptation, but rather may be local adaptation. There is a room to explore that. The question is: is introgression adaptive only in the direction from arenosa to lyrata (for adaptation to WGD), or can it also be adaptive in the opposite direction (maybe local adaptation)?

Reviewer #2 (Remarks to the Author):

This manuscript studied the molecular basis of parallel adaptation to WGD in lyrata compared to arenosa. In order to address this question, authors used 92 genome sequences from 92 individuals of lyrata, arenosa and outgroups, and cytologically assessed meiotic stability and selective sweep analysis and looked the overlap between sweep and introgression. Overall, authors suggested that the molecular basis by which WGD was stabilised in lyrata and arenosa is shared, and the whole story is generally interesting.
Comments and suggestions:
1) I am not sure it is reasonable to say rescued the nascent A. lyrata tetraploids from early extinction, such as in abstract. At least, so far we do not have enough evidence to claim a newly originated tetraploids is easy to extinct because of meiotic stability. Authors might need to rephrase somehow.
2) Besides meiotic stability related genes, from the selection sweep analysis, authors must have found other genes, it will be great to characterize those genes as well, which must be very important for the polyploidy's survive as well.
3) Again, in the introgression section, besides meiotic stability related genes, what are the other genes? Which kinds of GO terms authors enriched? These are all important detailed information needed for the story
4) 1% outliers from genome scan, this has to be explained more carefully, it would be great to perform some additional permutation to support this cut-off, or to explain this in detail somehow.

Reviewer #3 (Remarks to the Author):

The manuscript by Marburger et al titled "Interspecific introgression mediates adaptation to whole genome duplication" describes analyses of genome sequences from diploid and tetraploid A. lyrata and A. arenosa individuals. This study investigates patterns of divergence between ploidy levels and species, signatures of selection associated with ploidy increase, and signatures of introgression between the two species. It concludes that an overlapping set of genes are under selection in tetraploids of the two species. These genes are also enriched for signatures of introgression between the species.

Although this paper tells a nice story, my concern is that it is an incremental contribution to our understanding of ploidy evolution in Arabidopsis. The authors re-do many of the same analyzes they previously did in A. arenosa on A. lyrate and, maybe not surprisingly, find similar patterns. Analyses of introgression between these species have been done and it is widely accepted that hybridization is very common in sympatric plants and often results in adaptive introgression so I'm not sure that this finding sufficiently novel. I also found the patterns of direction of gene flow a bit unclear. Below are

more specific comments about the paper (although it is difficult to refer to locations without page numbers).

I think there is some excessively strong language that is unnecessary and distracting for example in the intro and abstract: "escape from extinction following the trauma of WGD.."
"evolutionary genomics underscores the ubiquity of WGD"
" WGD and hybridization are dramatic mutations"

In the population structure analysis, is there an explanation for why the outgroup "CRO" groups with arenosa? With 4 clusters does this separate out?

Given the variation in admixture and the strong geographic structure to these populations it would be helpful to have a map that labels these populations or at least shows how the populations are grouped (LET, LWT). It is implied that admixture proportion is related to geographic proximity between tetraploids but it would be nice to have this be tested and to visually see what the patterns look like. Looking at reference 22 (Hohmann and Koch) I believe the pattern of introgression will be similar to figure 1. Is there anything new about the patterns of introgression in this study compared to that study?

The authors seem to imply that polyploidization itself allowed for introgression between the two species but it seems that the geographic range overlap is more likely responsible for determining the extent of geneflow between species. It doesn't look like the diploids overlap in range and therefore they don't hybridize. The tetraploids do overlap and the more they overlap the more they hybridize. Is there any evidence that the WGD actually directly enabled or allowed for introgression?

It's unclear to me where the estimates of gene flow (alleles/generation) in the text come from as the figure (S2) and table (S2) have different numbers. All the other numbers suggest asymmetric gene flow from lyrata into arenosa but the text reports symmetric.

I am unclear on the DFOIL test that was performed. As I understand it, DFOIL works on a symmetric tree (in this case first split is between species and second splits are between populations in a species). Based on Figure S5, it looks like their trees are not symmetric and arenosa is not analysed as a monophyletic group. How was this analysis done? It's also unclear to me why these particular populations were chosen and why it is even interesting what the direction of gene flow is. Furthermore, if the gene flow is ancestral to the tetraploid split, it seems to make sense to use a tetraploid and diploid lyrata sample to determine direction of introgression with arenosa. I'd like to see a more justified explanation for what the analysis is and why it was done and how it relates to the structure analysis and the fastsimcoal2 analysis. Why is there such a clear sign of directional geneflow in DFOIL but not with fastsimcoal2?

The authors find that meiosis remains unstable in lyrata. This instability segregates within populations. Is there any connection between the instability and the degree of introgression? Or the loci that are implicated in selection? Given the remaining instability in both species it seems that stability is still evolving and therefore selected alleles could have arisen recently in either of the two species and be spreading. It is unclear to me how the authors are interpreting the instability and how it fits into their story of adaptation and introgression after WGD.

For the selection outlier analysis, why were the top 1% of the lyrata outliers investigated but only the top 0.5% of the arenosa outliers look at? Is the pattern different if only the top 0.5% of the lyrata are considered? I would like to see more numbers in reporting of these outlier results. It is stated that "the majority of known loci" were also found in arenosa but what are the numbers? Are the selected loci in lyrata statistically enriched for meiosis/endopolyploidy/transcription genes as in arenosa and if so I'd like to see some statistics for this. When comparing the outliers from the Let and the Lwt analysis the authors state that there is considerable overlap. I'm not sure < 1/3 of the genes is

considerable overlap. It would be more effective to just state the overlap. It is also stated that "genes encoded by these loci include many involved in genome maintenance and meiosis" but I would like to know what those numbers are as well.

I would appreciate a clearer explanation of the Twisst results. It is stated that there are three introgression-indicative topologies (3, 11,14) but 3 is the dominant species topology and is not consistent with introgression so this needs to be clarified or corrected. It is unclear how this topology based inference controls for incomplete lineage sorting (which is likely very common in these new lineages). Their narrow windows of introgression are even more suggestive of ILS. Another test based on allele divergence as well as topology would likely be a better indication of which regions are introgression and which ILS. The summary of the Twisst results are confusing to me as well. The authors state that "several meiosis-related" loci are outliers in Twisst and list 4 then state that "only 4" are not outliers for twist but divergence. These are the same number of loci but discussed with different adjectives. The next sentence states that "the vast majority of these loci were indicated as undergoing specific gene flow". This statement is again inconsistent with the numbers above. I think these numbers need to be more clearly and fairly stated.

The overall conclusion of the direction of geneflow is very unclear to me. It seems that the Fastsimcol2 results show more lyrata to arenosa gene flow, the Dfoil shows symmetric or more arenosa to lyrata gene flow and the Twisst analysis shows marginally more lyrata to arenosa gene flow. Yet, the authors conclude that there is adaptive introgression of alleles from arenosa to lyrata. I think there is no evidence that this should be the conclusion. The story about adaptive alleles for stabilizing WGD is even more unclear because the authors argued that some of these alleles came from the diploid populations (see figure 2). Together these inconsistencies point out how tenuous it is to make functional conclusions based on population genetic sequence data alone.

In summary, the authors find evidence of selection in the genome of A. lyrata. Some of these outlier loci appear to be involved in meiosis and could be implicated in stabilization after WGD. This is exactly what was found in A. arenosa with the same analyses. The authors find evidence of introgression between the tetraploids in the same way that previous studies have found introgression. The introgression is likely bi-directional but the tests are inconsistent. Some regions showing discordant gene-tree topologies also contain regions that are outliers for selection. The major inconsistencies in their data include the direction of gene flow as indicated by the different tests, the timing of geneflow relative to polyploidization and genome stabilization, and the function of the introgression in actually stabilizing meiosis after WGD.

The
Figures:
Fig. 1. Please explain your abbreviations in part A. It would be helpful to have the ploidy level indicated in part B (for example with different shapes) to clearly see how species and ploidy divide along the PC's. It would also be helpful to see what the populations are that are later called LET and LWT. In part C the description says the lines are proportional to parameters in Fig S2 but the migration estimates (M5 and M6) are different in S2 but look the same in the manuscript figure.

Fig. 2. I would like to see a scale bar on part B. It is also impossible to read the top axis.

Fif 3. I would like a clearer description of what the "weighting" axis is in part C. It should also be stated that the colors in part B and C correspond to the colors of the topologies in part A. I think it should be explained why the 4 of the top 5 topologies were not looked at (top 10, 15, 2, 1).

Reviewer #1 (Remarks to the Author):
Marburger at el. describes a population genomic study in which they test the hypothesis that adaptive gene flow (of genes adapted to increase meiotic stability and/or reduce meiotic crossovers) from one species to another mediates escape from extinction following WGD. In particular, the authors show that specific pre-adapted alleles 'donated' from tetraploid Arabidopsis arenosa to tetraploid Arabidopsis lyrata (which has undergone a WGDmore recently than arenosa) probably rescued the nascent A. lyrata tetraploids from early extinction.

I have read this paper with great interest, but have the following comments.

The authors mention several times that the pre-adapted alleles donated from A. arenosa to A. lyrata are responsible for rescuing A. lyrata from extinction. However, to this reviewer it is unclear whether tetraploid A. lyrata would indeed have gone extinct without adaptive introgression of alleles from A. arenosa or just adapt to WGD more slowly? Is there any way to 'proof' that, if not having obtained these adapted alleles by gene flow, the new tetraploids would have gone extinct? Is this suggested by the paragraph 'stabilisation of lyrata meiosis following WGD', where the authors mention that meiotic stability is segregating within tetraploid populations? This is not entirely clear to me. I have the feeling the authors might be more careful in phrasing this. I think the observation that pre-adapted genes have been obtained by gene flow is very interesting and for sure are a strong indication that these helped A. lyrata coping with the WGD (as has been shown previously for A. arenosa), but this is not the same as assuming that otherwise tetraploid A. lyrata should have gone extinct for sure. If true, then, we also need to assume that A. arenosa would have received its 'adapted' genes from somewhere else (or it too might have gone extinct ..).

We thank the reviewer for these helpful suggestions and agree strongly. We have reworked the text accordingly. Please see the highlighted changes in the text in the abstract (e.g., removing both times we suggested these data indicate that nascent A. lyrata would have gone extinct without these alleles). We have removed all instances from the manuscript that suggest that these alleles rescued the newly claimed tetraploids from extinction.

The authors state that '… selective sweep signatures directly overlapping eight loci that interact during prophase I..'. I assume that it is not the genetic loci that directly interact but rather their gene products?

Yes, thank you. We have reworked the text to state this correctly. Please see the change on page 3.

The authors state that they '… estimated the age of WGDs at 81k generations for lyrata (~160,000 years)'. In this respect, I think the following papers could be cited as well (Hohmann and Koch, 2017; Novikova et al., 2018).

Thank you for the addition of Hohmann and Koch; we added this to the position on page 6. As we read it, Novikova cites Hohmann and Koch for her date in her review, so we only have added Hohmann and Koch.

The authors state that interspecific gene flow from tetraploid arenosa to tetraploid lyrata (0.0998 alleles/generation) and lyrata to arenosa are at the same level (0.1001 alleles/generation)"
How come arenosa->lyrata flow is lower than lyrata->arenosa? Admixture patterns and Dfoil results suggest the opposite, or am I missing something here?

Our initial presentation of these several lines of evidence was unclear. In light of this and the other reviewers' comments, we have carefully revisited all analyses of gene flow and their presentation. Most

importantly we realised that Dfoil was underpowered and operated in windows too large in this analysis. Overall, the Dfoil results were much less consistent and less convincing than the results of fastsimcoal2 *and Twisst. Thus, we have removed the Dfoil analysis entirely. This leaves a more consistent analysis that better represents the whole of the data. Also, for simplicity, a small point: the retention of too many significant digits in the estimates above was also distracting, so we appropriate report both directions as 0.1 alleles/generation now. Please see our revised presentation on pages 7, and 12-14, which we believe much more clearly describes the approximately even levels of bidirectional gene flow that we can localise to many of the genes harbouring the strongest signals of selective sweep.*

The authors experimentally show that meiotic stability is varying from population to population (Table S3), but they fail to show if it depends on the presence of particular sets of adaptive alleles (from Table 2, for example) or admixture assignments. Is this something that could be looked at?

Yes, we agree this is a great idea and an important future direction. In fact, the lab of James Higgins, a co-author on this manuscript, is leading such a study. We would love to include all of this in one very big manuscript, but we encountered the issue that carefully scoring meiotic stability against the presence of particular alleles is very laborious, especially in an autotetraploid, where four alleles must be cloned and Sanger sequenced at every locus for phased haplotype assessments in a large number of individuals for statistical robustness. These studies are ongoing, but they require investment well beyond the scope of this current report.

In general, I think it would be nice if the authors could briefly explain the population statistics they used (I, for example had to look up Rho, and there are several others like this for which I'm not sure the average reader will know what the authors mean with these).

Thank you for asking. We have added some more description and references for all. Please see on pages 8 and 9:

Page 8: "… d_{XY}[28], Fst[29], and Rho[30] … Fst is influenced by within population diversity, and lacks sensitivity in cases of low differentiation. Therefore, we used additional differentiation metrics. dXY does not take within population diversity into account, whereas Rho is a divergence measure that is independent of ploidy level and double reduction in autopolyploids." and "Multiple differentiation metrics were used, as the metrics exhibit different sensitivities to diversity and differentiation. Values of all metrics were averaged over pairwise comparisons of populations belonging to that group."

The authors state that 'Overall, genome-wide differentiation between lyrata diploids and the tetraploid populations was extremely low (mean Rho between ploidies = 0.19)'. Low compared to what? It is the highest value in the table?! Table 1 needs the same statistics for diploid vs. tetraploid A. arenosa. I think this is what they compare it with?

We expressed what we wanted to say very poorly: thank you for catching this. Stating it that way says something different from our intention. We simply meant to describe the shallow divergences between groups in the study generally.

We rephrased page 8 as "Overall, genome-wide differentiation levels between lyrata diploids and the tetraploids indicate shallow divergence between all groups (with mean Rho in the most differentiated contrast between ploidies = 0.19; Table 1; Supplemental, Table 4 for additional population contrasts), consistent with our previous studies in arenosa[12,13,21,31]"

Fig. 2A should be better explained. What is the red line? There are two y-axes, however, which one is which (dots/line)?

We agree completely and apologise for the insufficient legend. Please see the revised version.

'..tetraploids, Lwt hereafter: PIL, SCB, KAG, SWA, LOI, MAU). 'What happened to the GYE population? It is included in the STRUCTURE analysis but not in any of the scans. Or am I missing something?

We should have explained why we excluded GYE from some analyses. This was because it is part of a very different biogeographic region (Pannonia). We were conservative in scans for selective sweep in that we wanted to restrict our diploid vs. tetraploid comparison to populations from the same biogeographic region (eastern Austrian Forealps and nearby Wachau). We have added an explanation for this on page 9: "GYE was excluded due to distant geographic grouping in Pannonia".

Figure 3 shows clear signal for introgression from lyrata to arenosa (topo 11) as well, which doesn't overlap with genome scans and is probably not involved in WGD adaptation, but rather may be local adaptation. There is a room to explore that. The question is: is introgression adaptive only in the direction from arenosa to lyrata (for adaptation to WGD), or can it also be adaptive in the opposite direction (maybe local adaptation)?

We agree this is an interesting suggestion. We would love to focus on exploring such patterns. In fact, we saw related patterns between ploidies specifically in A. arenosa *in our recent manuscript (Monnahan et al, 2019, Nature Ecology and Evolution). At your suggestion we did a scan for this among the loci here that Lwt and Let selection outliers as Twisst introgression positive and unfortunately, we did not pick up loci with known robust functional descriptions beyond those we highlight already, so we don't highlight them in the main text (for the interested reader who would like to dig deeper of course all locations, Twisst signals and gene names/descriptions are given in Supplemental Dataset 3). We also now report the GO enrichment we found among these genes under selection and with strong evidence of gene flow on page 14.*

Reviewer #2 (Remarks to the Author):

This manuscript studied the molecular basis of parallel adaptation to WGD in lyrata compared to arenosa. In order to address this question, authors used 92 genome sequences from 92 individuals of lyrata, arenosa and outgroups, and cytologically assessed meiotic stability and selective sweep analysis and looked the overlap between sweep and introgression. Overall, authors suggested that the molecular basis by which WGD was stabilised in lyrata and arenosa is shared, and the whole story is generally interesting.

Thank you very much for the encouraging comments.

Comments and suggestions:
1) I am not sure it is reasonable to say rescued the nascent A. lyrata tetraploids from early extinction, such as in abstract. At least, so far we do not have enough evidence to claim a newly originated tetraploids is easy to extinct because of meiotic stability. Authors might need to rephrase somehow.

We agree completely. Please see the highlighted changes in the text in the abstract (removing both times we stated this) and introduction especially. We have removed all instances from the manuscript that suggest that these alleles rescued the newly claimed tetraploids from extinction.

2) Besides meiotic stability related genes, from the selection sweep analysis, authors must have found other genes, it will be great to characterize those genes as well, which must be very important for the polyploids survive as well.

Here also we agree completely that such genes are important and merit greater emphasis. We were previously concerned about space and simplicity of presentation. However, we are happy to follow your suggestion. Accordingly, we have added the following analysis and results to the text on page 9:

"Gene ontology enrichment analysis of these 196 genes identified significant overrepresentations in categories related to meiotic and homologous chromosome segregation, but also diverse processes including epidermal cell differentiation, trichoblast maturation, root hair cell and epidermal differentiation, root hair cell development and elongation, and others such as indole-containing compound metabolic process and mRNA catabolic process (Supplemental Figure 5 and Supplemental Dataset 2). These results indicate that evolutionary change may occur throughout a broad array of processes during adaptation to WGD, beyond meiotic chromosome segregation."

This is of course in addition to our previous discussion on pages 11-12: "Apart from loci encoding meiosis-related genes, we detected extreme differentiation at loci belonging to other functional categories clearly related to the challenges attendant to WGD, including loci involved in endoreduplication and transcriptional regulation: CYCA2;3, PAB3, NAB, TFIIF, and GTE6. Whole genome duplication increases the ploidy of all cells, while endopolyploidy occurs in single cells during their differentiation, and this cell- and tissue-specific ploidy variation is important in plant development[33-35]. Thus, given the instantly doubled organism-wide nuclear content following WGD, we postulate that the degree of endopolyploidy would be modulated in response, with accumulating support for this notion[36-38]. Our findings bolster the idea that there may be a link between organism-wide polyploidization and that of single cells within an organism. Research about the effect of WGD-induced dosage responses of the transcriptome is still in its infancy[3,39], and emerging studies on allopolyploids support incomplete dosage compensation."

3) Again, in the introgression section, besides meiotic stability related genes, what are the other genes? Which kinds of GO terms authors enriched? These are all important detailed information needed for the story

Here we have also followed your advice but found only GO enrichment for meiosis in this set. We do discuss several other non-meiosis genes we see in this list, but there are not many in the set that are most highly selected and additionally show evidence of gene flow. Additionally, we give the complete list of Twisst-positive genes in Supplemental Dataset 3. In general, however, of loci with clear signatures of selection AND specific introgression signal by Twisst, it really appears to be overwhelmingly focussed on meiosis. Please see page 14: "In addition to meiosis related genes we do see introgression signal at the endopolyploidy gene CYCA2;3 and the global transcriptional regulator TFIIF, but very few other loci exhibit both persistent signatures of extreme selection as well as introgression (Supplemental Dataset 3)."

4) 1% outliers from genome scan, this has to be explained more carefully, it would be great to perform some additional permutation to support this cut-off, or to explain this in detail somehow.

We agree that as presented these cut-offs seem arbitrary on their face. Our reasoning was that comparing multiple sets of outliers determined with hard cut-offs misses differentiated loci that stochastically fall just below such cut-offs in any given scan. Importantly, we overlaid the results of two replicate genome scans in lyrata *and so we used a slightly relaxed cut-off for each of our two constituent sets of empirical outliers that we then stringently required the overlap. In other words, in the end we note that we do not require a locus to be a 1% outlier in a single scan (e.g. Let or Lwt), but in fact it must be a 1% outlier in* ***both*** *independent scans *as well* as an outlier in the 2013 arenosa scan, considerably increasing the stringency of the scans (even Let/Lwt double outliers number only 196 out of a total of 31,132 gene models in our annotation (19600/31132=0.6% of the total)*

We have added some modifications of the text at page 8 to explain our reasoning right upfront to frame the divergence scan better: "To identify the most robust signals of selection in the tetraploid lyrata populations, we performed genome scans on two different lyrata tetraploid population groups and then focussed on the genes that were repeatedly in the extreme 1% outlier windows in both contrasts. This identified 196 genes, (0.6% of gene-coding loci in the genome; Supplemental Dataset 1)."

Regarding the idea for permutation to support this cut-off, in principle this can be attractive, and we have tried this in the past, but our experience with such simulations has been fraught with problems. For example, lacking sufficient knowledge of the true recombination and fine-scale demographic landscapes in sufficient detail gives noisy, unrealistic estimation of realistic cut-offs in our system. Instead we believe requiring a locus to be a moderately strong empirical outlier in two within species scans gives us more confidence, with the final overlap with the arenosa divergence scan of 2013 giving twenty loci that have conspicuous and convincing gene functions and divergence scan profiles (e.g. Table 2 and Figure 2). In fact, we strongly believe this is a very conservative approach, which likely suffers from more false negatives than false positives.

Reviewer #3 (Remarks to the Author):
The manuscript by Marburger et al titled "Interspecific introgression mediates adaptation to whole genome duplication" describes analyses of genome sequences from diploid and tetraploid A. lyrata and A. arenosa individuals. This study investigates patterns of divergence between ploidy levels and species, signatures of selection associated with ploidy increase, and signatures of introgression between the two species. It concludes that an overlapping set of genes are under selection in tetraploids of the two species. These genes are also enriched for signatures of introgression between the species.
Although this paper tells a nice story, my concern is that it is an incremental contribution to our understanding of ploidy evolution in Arabidopsis. The authors re-do many of the same analyzes they previously did in A. arenosa on A. lyrate and, maybe not surprisingly, find similar patterns. Analyses of introgression between these species have been done and it is widely accepted that hybridization is very common in sympatric plants and often results in adaptive introgression so I'm not sure that this finding sufficiently novel. I also found the patterns of direction of gene flow a bit unclear. Below are more specific comments about the paper (although it is difficult to refer to locations without page numbers).

Thank you for your frank comments. While we appreciate your impression and find it valuable in our revision.

I think there is some excessively strong language that is unnecessary and distracting for example in the intro and abstract: "escape from extinction following the trauma of WGD.."
"evolutionary genomics underscores the ubiquity of WGD"
" WGD and hybridization are dramatic mutations"

We agree. Our language tended to be unnecessarily strong and we don't wish for it to distract. We have toned it down overall. (e.g. striking all instance of 'escape from extinction'; changing 'ubiquity' to 'high frequency'; 'dramatic mutations' to 'large effect mutations'; 'crucial' to 'essential'; deleting words like 'conspicuous') Please see highlighted changes e.g. on pages 2-4.

In the population structure analysis, is there an explanation for why the outgroup "CRO" groups with arenosa? With 4 clusters does this separate out?

Arabidopsis croatica *is indeed closely related to arenosa (see Hohmann et al., 2014; Novikova et al., 2016), yet sufficiently differentiated from it. We add mention (page 4) that it is a separate species. In the Twisst analysis, which requires an outgroup, we used* Arabidopsis halleri*, which is in sister relationship to arenosa/lyrata (Novikova et al., 2016).*

Given the variation in admixture and the strong geographic structure to these populations it would be helpful to have a map that labels these populations or at least shows how the populations are grouped (LET, LWT). It is implied that admixture proportion is related to geographic proximity between tetraploids but it would be nice to have this be tested and to visually see what the patterns look like. Looking at reference 22 (Hohmann and Koch) I believe the pattern of introgression will be similar to figure 1. Is there anything new about the patterns of introgression in this study compared to that study?

We followed a different sampling strategy compared to Hohmann and Koch in that we focused on the Wienerwald with both diploid and autotetraploid lyrata, the Wachau with largely autotetraploid lyrata, and the eastern Austrian Forealps with the intermediate hybrids between arenosa and lyrata; we sampled up to 70% more populations (in the case of the Wachau) in these regions compared to Hohmann and Koch.

Because of denser sampling, we found an increased representation of "pure" lyrata in the Wachau, whereas Hohmann and Koch had picked two of the more introgressed populations at the margins of the Wachau. Also, based on our denser sampling in the Forealps we found out that there is a hybrid zone there, which is a different scenario compared to the Wachau.

The fine-scale spatial role in introgression between arenosa and lyrata we believe is beyond the scope of this study and additionally will greatly benefit by much more detailed sampling. This work is ongoing by lead by the co-author Roswitha Schmickl. Thus, in this manuscript we much prefer to leave this aspect with an implication rather than a test that lacks robustness. Work as you suggest that focusses on this is in progress with denser sampling of populations and individuals per populations will prove more robust than a side note here, but we definitely see the value in your suggestion.

The authors seem to imply that polyploidization itself allowed for introgression between the two species but it seems that the geographic range overlap is more likely responsible for determining the extent of geneflow between species. It doesn't look like the diploids overlap in range and therefore they don't hybridize. The tetraploids do overlap and the more they overlap the more they hybridize. Is there any evidence that the WGD actually directly enabled or allowed for introgression?

We have added explicit reference to experimental justification for our implication that polyploidization itself allowed for introgression between the two species, and not geographic range: Lafon-Placette et al. (2017) (reference 20) characterise an endosperm-based postzygotic barrier when crossing diploids of both species, and they also demonstrated that this barrier is greatly relaxed in interspecific crosses of tetraploids, thus directly indicating ploidy level and not range overlap. Geographic vicinity of tetraploid arenosa and lyrata then increases the likelihood of gene flow.

We now added explicit mention of this study and the "endosperm-based postzygotic barrier" on page 16.

It's unclear to me where the estimates of gene flow (alleles/generation) in the text come from as the figure (S2) and table (S2) have different numbers. All the other numbers suggest asymmetric gene flow from lyrata into arenosa but the text reports symmetric.

Thank you for pointing out how that was very unclear: our presentation should have been much easier to understand. Alleles/generation must be multiplied by Ne in each lineage to get these estimates (per haploid genome). We have clarified our presentation and explained this in the relevant legends.

*The reason gene flow values for model 4 (the final chosen model) differed between Table S2 and Figure S2 was given (though easy to miss, we admit) in the Table S2 legend: "*Mean values are given, in contrast to medians in Figure 1C and Figure S2*" We now clarify our reasoning in addition: "*Mean values are given, in contrast to medians in Figure 1C and Figure S2, in order to get an estimate across replicates. Gene flow estimates must be multiplied by Ne for alleles/generation per haploid genome.*"*

I am unclear on the DFOIL test that was performed. As I understand it, DFOIL works on a symmetric tree (in this case first split is between species and second splits are between populations in a species). Based on Figure S5, it looks like their trees are not symmetric and arenosa is not analysed as a monophyletic group. How was this analysis done? It's also unclear to me why these particular populations were chosen and why it is even interesting what the direction of gene flow is. Furthermore, if the gene flow is ancestral to the tetraploid split, it seems to make sense to use a tetraploid and diploid lyrata sample to determine direction of

introgression with arenosa. I'd like to see a more justified explanation for what the analysis is and why it was done and how it relates to the structure analysis and the fastsimcoal2 analysis. Why is there such a clear sign of directional geneflow in DFOIL but not with fastsimcoal2?

As a result of the reviewers' comments, we have carefully reassessed our several methods for gene flow estimation. As a result, we have kept fastsimcoal2 and Twisst, but realised that the DFOIL analysis was underpowered given our SNP density and the large window sizes it requires, so we therefore believe it gave overly noisy and inaccurate estimates, confusing the analysis further.

In response to the reviewer's question 'why it is even interesting what the direction of gene flow is,' we would say that it is very interesting in this case that our results here indicated that a roughly even mixture of these functionally and physically interacting prophase I meiotic crossover-mediating genes appear to have originated in each of these two diploids, coming together to all be selected strongly in the autotetraploids. We believe that this description of bidirectional adaptive gene flow is quite novel and surprising. Prompting us to wonder: why would it not be unidirectional, given that these proteins must interact functionally and physically?

The authors find that meiosis remains unstable in lyrata. This instability segregates within populations. Is there any connection between the instability and the degree of introgression? Or the loci that are implicated in selection? Given the remaining instability in both species it seems that stability is still evolving and therefore selected alleles could have arisen recently in either of the two species and be spreading. It is unclear to me how the authors are interpreting the instability and how it fits into their story of adaptation and introgression after WGD.

We absolutely agree this is a great idea and an important future direction, which reviewer #1 also mentioned. As we replied above to them: "In fact, the lab of James Higgins, a co-author on this manuscript, is leading such a study. We would love to include all of this in one very big manuscript, but we have run into the issue that carefully scoring meiotic stability against the presence of particular alleles is very laborious, especially in an autotetraploid, where four alleles must be cloned and Sanger sequenced at every locus for haplotype assessments in a large number of individuals for statistical robustness. These studies are ongoing, but they are requiring investment well beyond the scope of this current study."

For the selection outlier analysis, why were the top 1% of the lyrata outliers investigated but only the top 0.5% of the arenosa outliers look at? Is the pattern different if only the top 0.5% of the lyrata are considered? I would like to see more numbers in reporting of these outlier results. It is stated that "the majority of known loci" were also found in arenosa but what are the numbers? Are the selected loci in lyrata statistically enriched for meiosis/endopolyploidy/transcription genes as in arenosa and if so I'd like to see some statistics for this. When comparing the outliers from the Let and the Lwt analysis the authors state that there is considerable overlap. I'm not sure < 1/3 of the genes is considerable overlap. It would be more effective to just state the overlap. It is also stated that "genes encoded by these loci include many involved in genome maintenance and meiosis" but I would like to know what those numbers are as well.

We agree that as presented these cut-offs seem arbitrary on their face. Our reasoning was that comparing multiple sets of outliers determined with hard cut-offs misses differentiated loci that stochastically fall just below such cut-offs in any given scan and we wanted the most robust outliers in multiple scans. Thus, we overlaid the results of two replicate genome scans in lyrata*, so we used a slightly relaxed cut-off for each of our two constituent sets of empirical outliers that we then required the strict union.*

In other words, in the end we note that we do not require a locus to be a 1% outlier in a single scan (e.g. Let or Lwt), but in fact it must be a 1% outlier in ***both*** *independent scans *as well* as an outlier in the 2013 arenosa scan, considerably increasing the stringency of the scans (even Let/Lwt double outliers number only 196 out of a total of 31,132 gene models in the reference annotation (19,600/31,132=0.6% of the total)*

We have added some modifications of the text at page 8 to explain our reasoning right upfront to better frame the divergence scan section: "To identify the most robust signals of selection in the tetraploid lyrata populations, we performed genome scans on two different lyrata tetraploid population groups and then focussed the genes that were repeatedly in the extreme 1% outlier windows in both contrasts. This consisted of 196 genes, (0.6% of gene-coding loci in the genome; Supplemental Dataset 1)."

As the reviewer suggests, we have also clarified our statement about finding a 'majority' of known loci on page 9: "We observed selective sweep signatures at the majority (6/11) of coding loci of known function that were found as the very top outliers in arenosa (0.5% outliers for all three metrics used in that study)"

Regarding the Let/Lwt overlap of 1/3, we agree with the reviewer: we would much rather not overplay our language. Therefore, we take the reviewer's advice and simply state the extent of overlap, allowing the reader to make their own judgement.

Finally, following the reviewers' suggestions we have performed GO analyses in several relevant parts of the manuscript to give the appropriate statistics, which strengthen the story. These are on pages 9. 10 and 14, as well as in new Supplemental dataset 2.

I would appreciate a clearer explanation of the Twisst results. It is stated that there are three introgression-indicative topologies (3, 11,14) but 3 is the dominant species topology and is not consistent with introgression so this needs to be clarified or corrected. It is unclear how this topology based inference controls for incomplete lineage sorting (which is likely very common in these new lineages). Their narrow windows of introgression are even more suggestive of ILS. Another test based on allele divergence as well as topology would likely be a better indication of which regions are introgression and which ILS. The summary of the Twisst results are confusing to me as well. The authors state that "several meiosis-related" loci are outliers in Twisst and list 4 then state that "only 4" are not outliers for twist but divergence. These are the same number of loci but discussed with different adjectives. The next sentence states that "the vast majority of these loci were indicated as undergoing specific gene flow". This statement is again inconsistent with the numbers above. I think these numbers need to be more clearly and fairly stated.

We apologise for our typo in the description: In that instance, '3' should have read '6'. We have also considerably reworked the Twisst results section, including figure 3. Please see pages 12-14 for a much clearer extended discussion taking into account all of the reviewers' helpful points.

Regarding our overly dramatic language, we have worked hard to totally remedy this. For example, the issue at the end of the reviewer's comment (same number, different adjectives) we have rephrased as a more judicious: "we observe that four meiosis-related loci were outliers in all Twisst and divergence scans: PRD3, ASY3, SYN1, and DYAD (Supplemental Dataset 3), and four did not show a signal in both Twisst analyses as well as both divergence scans: ASY1, ZYP1a, ZYP1b, and PDS5."

Regarding ILS: we agree, it is always a challenge to distinguish ILS from introgression. However, the design here with a symmetrical tree and an outgroup means that we do have some power to evaluate the two hypotheses.

Under the hypothesis of ILS, we assume that the tetraploid-favoured allele was segregating alongside the diploid-favoured allele in the common ancestor. After speciation, both alleles must then have been retained in both species (i.e. ILS), and the evolution of tetraploids then led to the independent spread of the tetraploid-favoured allele. Under this model, only topology 6 is expected. Topologies 11 and 14 are both much less likely as the nested position of the tetraploid allele within diploid alleles implies that speciation preceded the emergence of the tetraploid allele. Given that most peaks are T11 and T14, introgression is the more likely hypothesis.

We take it that the basis of the suggestion the thin peaks suggest ILS is the idea that introgression involves recent events, whereas ILS is ancient. Large, intact shared tracts are best explained by recent introgression, because polymorphic alleles that survive speciation events (ILS) tend to be broken down by recombination and will not be very large. The problem with the argument is that there is no baseline, especially without a recombination map. How large do we expect the tracts to be? If introgression occurred long ago, there could be enough recombination that we would expect only the narrow beneficial tracts to be shared in tetraploids today.

In fact, we have evidence that these narrow tracts might be able to form rapidly in these autotetraploids: we have recently published a linkage estimate specifically in 182 tetraploid A. arenosa genomes from 24 populations compared with diploids from the same species (Monnahan et al 2019, Nature Ecology and Evolution: blue lines in figure on right represent linkage per autotetraploid population from that publication). This dramatic reduction in genotypic correlations relative to diploids suggest a mechanism by which sharp peaks of divergence may form rapidly in these autotetraploids.

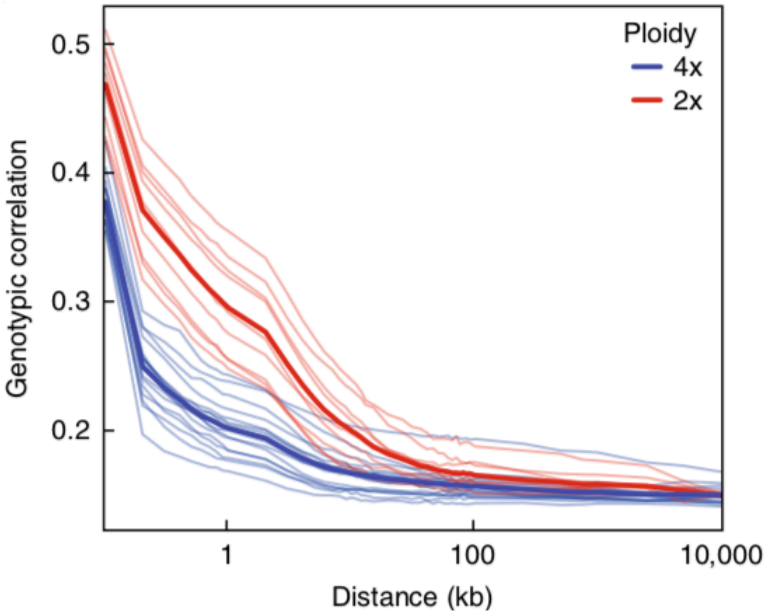

Decay of genotypic correlations within each population and averaged for each ploidy (heavy lines) as a function of distance between sites (from Monnahan et al 2019)

Beyond this, we would mention that we have uniformly observed in our several genome scans for selective sweeps in these autotetraploids that sweeps are single-gene in width, without any suggestion of ILS (see also e.g. our study in this system of serpentine adaptation: Arnold et al PNAS 2016, reference 31) Moreover, if there is gene conversion in these species, that could allow introgression of a very narrow tract indeed, even if it occurred recently.

We would note another line of evidence for gene flow over ILS in these data: the other non-species-tree-like topologies do not show similar clear peaks across the genome. If this was simply ILS without selection, we would expect to see peaks of other topologies such as ((arenosa_tet, lyrata_dip),(arenosa_dip, lyrata_tet)). The absence of such peaks implies selection for shared alleles between tetraploids specifically.

The overall conclusion of the direction of geneflow is very unclear to me. It seems that the Fastsimcol2 results show more lyrata to arenosa gene flow, the Dfoil shows symmetric or more arenosa to lyrata gene flow and the Twisst analysis shows marginally more lyrata to arenosa gene flow. Yet, the authors conclude that there is adaptive introgression of alleles from arenosa to lyrata. I think there is no evidence that this should be the conclusion. The story about adaptive alleles for stabilizing WGD is even more unclear because the authors argued that some of these alleles came from the diploid populations (see figure 2). Together

these inconsistencies point out how tenuous it is to make functional conclusions based on population genetic sequence data alone.

Our sincere apologies for that. Given the confusion we allowed around the presentations of the fastsimcoal *results, we can see how this was difficult for the attentive reviewer. We have simplified the revised version and removed the Dfoil test, which we believe upon re-inspection to have been underpowered for this data set, despite the high density of the data. All results from Twisst and* fastsimcoal2 *consistently indicated approximately even gene flow in both directions. Further, Twisst gives very clear pictures of exact loci that likely originate in one or other diploids species that we find now to be under extreme selection in both species. We saw also in Yant et al 2013 and studies since that adaptive alleles can indeed be present as standing variation in diploids so these findings of indicated origin in one or another of the diploid species are reasonable here.*

In summary, the authors find evidence of selection in the genome of A. lyrata. Some of these outlier loci appear to be involved in meiosis and could be implicated in stabilization after WGD. This is exactly what was found in A. arenosa with the same analyses. The authors find evidence of introgression between the tetraploids in the same way that previous studies have found introgression. The introgression is likely bi-directional but the tests are inconsistent. Some regions showing discordant gene-tree topologies also contain regions that are outliers for selection. The major inconsistencies in their data include the direction of gene flow as indicated by the different tests, the timing of geneflow relative to polyploidization and genome stabilization, and the function of the introgression in actually stabilizing meiosis after WGD.

We wonder whether this summary reflects a combined effect of our unfortunately quite confusing presentation from our initial submission and we regret this. Thanks to this feedback, we believe we have now greatly clarified the text to protect against such readings and represent the analysis better. Importantly we have removed the confusing and underpowered Dfoil analysis and have cleaned up the presentation of the fastsimcoal2 results, as well as the Twisst presentation.

*We are disheartened to think we might have produced an impression to generate the reviewer's comment: "*The major inconsistencies in their data… include the timing of geneflow relative to polyploidization and genome stabilization, and the function of the introgression in actually stabilizing meiosis after WGD*" But, respectfully, we do not agree. Regarding the first point: the gene flow timing is not precisely determined (nor can it be). That standing genetic variation in the diploids may have produced alleles that were later selected in the young tetraploids is not at all inconsistent. Regarding the second: the function of the introgressed alleles is very clear. These is an obvious statistical enrichment of genes that mediate crossover number and physical distribution in prophase I of meiosis (we hypothesise by modulating the strength of crossover interference) that we have first revealed in Yant et al 2013 in arenosa. That these functionally and physically interacting genes have origins in *different* diploid species, coming together in the autotetraploids and from there spreading to both autotetraploid species, we believe is an important and interesting point. We are sorry this was not clear enough in the previous version.*

Finally, we are happy to report a further novelty that we had wished to emphasise in the original submission but left out for simplicity but has emerged from the review process: namely that we discuss further processes under selection in these several scans for selection beyond simply meiosis (which was the sole focus of the 2013 arenosa study, subsuming even the title).

The Figures:
Fig. 1. Please explain your abbreviations in part A. It would be helpful to have the ploidy level indicated in part B (for example with different shapes) to clearly see how species and ploidy divide along the PC's. It

would also be helpful to see what the populations are that are later called LET and LWT. In part C the description says the lines are proportional to parameters in Fig S2 but the migration estimates (M5 and M6) are different in S2 but look the same in the manuscript figure.

Thank you for the suggestions. Figure 1A lists 30 populations (too many to define in the figure as a list); we now refer explicitly to Supplemental Table 1 that gives details of all of these.

*Good idea! We have now indicated ploidy level in 1B (diploids with grey outline) and indicated LET and LWT groupings. "*Diploids are indicated by grey outline. Asterisks (*) are placed under the *Let* diploid grouping; all other lyrata diploids (except the geographically divergent Pannonian GYE) are in the *Lwt* group.*"*

Thank you. Please see our comments above regarding the estimates of gene flow in the text vs figure (S2) and table (S2). The estimates from fastsimcoal2 are equal levels in each direction among tetraploids specifically and thus are appropriately weighted in the main figure. Again, our kind apologies for that confusion.

Fig. 2. I would like to see a scale bar on part B. It is also impossible to read the top axis.

We agree there was too much information in the top axis to print legibly; we had tried to list all 44 nonsynonymous coding changes in the figure. Please see the new version on page 2. We have cleaned up the presentation now and better indicate what is pictured in the legend and figure panels (e.g. scale given for part B: "PRD3 missense polymorphism frequencies. Range: amino acids 6 to 406"). In case the reviewer means by 'scale bar' a scale for the colours indicating allele frequencies, we add that as well to the right.

Fif 3. I would like a clearer description of what the "weighting" axis is in part C. It should also be stated that the colors in part B and C correspond to the colors of the topologies in part A. I think it should be explained why the 4 of the top 5 topologies were not looked at (top 10, 15, 2, 1).

Thank you for the suggestion; we agree the weighting should be better described. We have added the following to the Figure 3 legend: "The weighting quantifies the extent to which each 50 SNP window tree matches a given topology, accounting for the fact that each taxon is represented my multiple individuals that each have 2 (for diploids) or 4 (for tetraploids) tips in the tree. A weighting of 1 indicates that all individuals cluster in the same way, such that all possible sub-trees match the same topology. Weightings >0 but <1 indicate that different subtrees match different topologies."

Thank you for the good idea to state the colour consistency in the panels. We do this now.

Thank you for the suggestion: the objective here was to illustrate the highly specific regions exhibiting signals of gene flow and selective sweep. Thus, we focus only at the introgression-indicative topologies and found considerable overlap with the loci that are under the highest levels of selection in arenosa and lyrata. We modified the legend accordingly.

In summary, we really do appreciate your highly attentive, critical review. This has prompted us to very carefully address your points and we truly believe this has greatly improved the coherence of our manuscript.

REVIEWERS' COMMENTS:

Reviewer #1 (Remarks to the Author):

I think the authors have done their utmost best to address all my concerns (as well as those of the other reviewers, which I looked at as well), and I have no further comments.

Reviewer #2 (Remarks to the Author):

The revision addressed all my concerns, I am satified with the updated manuscript.

REVIEWERS'　COMMENTS:

Reviewer #1 (Remarks to the Author):

I think the authors have done their utmost best to address all my concerns (as well as those of the other reviewers, which I looked at as well), and I have no further comments.

Thank you for your constructive input over the course of the review and revision process.

Reviewer #2 (Remarks to the Author):

The revision addressed all my concerns, I am satified with the updated manuscript.

Thank you for your constructive input over the course of the review and revision process.
